# Uncovering the copper(i) binding abilities of a unique fungal metallothionein: characterization of *Yarrowia lipolytica* MT and its Y54C mutant

**DOI:** 10.1039/d5dt00689a

**Published:** 2026-04-13

**Authors:** Aleksandra Hecel, Mariano Briccola, Eva Freisinger

**Affiliations:** a Faculty of Chemistry, University of Wrocław 50-383 Wrocław Poland; b Department of Chemistry, University of Zurich 8057 Zurich Switzerland freisinger@chem.uzh.ch

## Abstract

Metallothioneins (MTs) are a superfamily of cysteine-rich proteins essential for metal homeostasis and detoxification. While mammalian and plant MTs have been extensively characterized, fungal MTs remain comparatively understudied despite their remarkable sequence diversity. In particular, members of the fungal-IV MT family from *Yarrowia lipolytica* exhibit unusual sequence features, including a conserved CCC motif and an extended cysteine-free N-terminal region, raising the question of whether all cysteine residues participate equally in Cu(i) coordination and how sequence context influences metal binding. To address this, we investigated one representative protein (Ylip_MT) using a combination of potentiometric titrations and complementary spectroscopic techniques to resolve the stepwise recruitment of cysteine ligands during Cu(i) coordination. Truncated variants were employed to probe the role of the cysteine-free N-terminal segment, while a Y54C mutant was introduced independently to restore the otherwise conserved C-terminal cysteine and assess its influence on metal binding. Our results indicate the formation of a Cu_4_Cys_8–9_ cluster and demonstrate that both the extended cysteine-free N-terminal region and the C-terminal environment modulate cluster stability. This work represents the first application of potentiometry to a Cu(i)-MT system and provides new insight into how sequence-specific features govern metal binding in fungal MTs.

## Abstract 2

Spectroscopic and potentiometric analyses show that *Yarrowia lipolytica* metallothionein hosts a Cu_4_Cys_8_ cluster with additional coordination of the N-terminal NH_2_ group.

## Graphical abstract

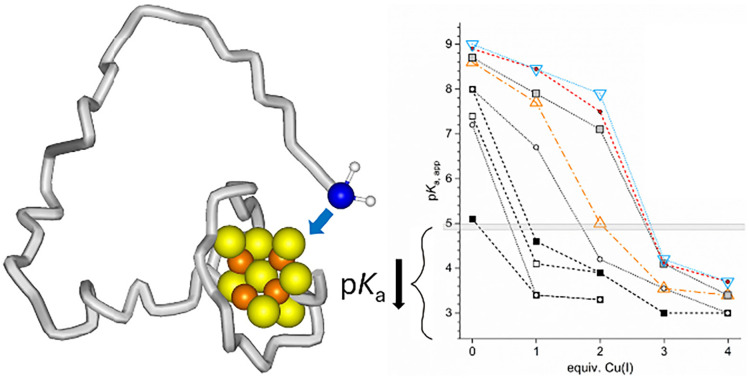

## 1. Introduction

Metallothioneins (MTs) constitute a superfamily of cysteine-rich proteins ubiquitous in nearly all living organisms. They play pivotal roles in maintaining homeostasis of essential metal ions, notably Zn(ii) and Cu(i), while also participating in the detoxification of non-essential heavy metal ions such as Cd(ii) or Hg(ii).^1–5^ Additionally, MTs are implicated in combating oxidative stress conditions.^4,6–9^ The coordination of metal ions primarily occurs through Cys thiolate groups, which additionally act as µ-bridging ligands to facilitate metal–thiolate cluster formation and efficient metal ion binding.^5,10^ Currently, MTs are classified into 15 families based on their amino acid sequences and phylogenetic relationships.^11,12^

In contrast to our understanding of mammalian and plant MTs, fungal MTs remain relatively understudied. They exhibit considerable diversity in primary amino acid sequences among different fungal species. Fungal MTs are grouped into six families, with each family comprising only a few or even a single member.^12^ Fungi, in the most common sense, that are diverse molds but also mushrooms, are classified under family fungi-I. Notably, yeasts, which also belong to the fungal kingdom, occupy the remaining five fungal families (*Candida glabrata*, fungi-II and -III; *Yarrowia lipolytica*, fungi-IV; *Saccharomyces cerevisiae*, fungi-V and -VI).

Copper is an essential trace metal ion in all living organisms^13,14^ but can also be cytotoxic due to its redox reactivity. In particular, Cu(i) can catalyse the production of free radicals in Fenton reactions,^15,16^ and disruption in copper homeostasis has been implicated in severe diseases such as Menke's and Wilson's diseases.^14,17–19^ MTs play a critical role by tightly binding Cu(i) ions, thereby shielding cells from oxidative stress.^20,21^

Most characterized fungal MTs belong to the Cu–thionein class, with the CUP1 protein from baker's yeast (*S. cerevisiae*) being one of the most extensively studied examples.^22,23^ Its NMR solution structure revealed coordination of seven Cu(i) ions within a single cluster,^24^ while single crystal X-ray crystallography identified an additional Cu(i) ion, resulting in a Cu_8_Cys_10_ cluster topology. This structure shows a combination of linear and trigonal-planar thiolate-only coordination of the copper ions.^25^ Of interest here are also two smaller fungal MTs from the family fungi-I, for which copper binding capacities were determined. The MT from *Agaricus bisporus* (portobello mushroom) contains seven cysteine residues and binds 5.8 equivalents of copper ions when isolated from its native source.^26^ A highly homologous MT from the filamentous fungus *Neurospora crassa* contains the same number of cysteine residues and incorporates six Cu(i) ions when cultivated in Cu(ii)-containing media.^27^ For comparison, the N-terminal β-domain of human MT3 shows an intermediate cysteine content of nine and is thus more similar to the protein investigated here (*vide infra*). It hosts the enigmatic Zn_3_Cys_9_ or Cd_3_Cys_9_ β-cluster but can also form well-defined major species containing four, six, or ten Cu(i) ions.^28,29^ Uniquely, the native form isolated from the human brain was found to contain a homometallic air-stable Cu(i)_4_–thiolate cluster in the β-domain, while the C-terminal α-domain hosts the enigmatic Zn_4_Cys_11_ α-cluster.^30–32^

**Fig. 1:**
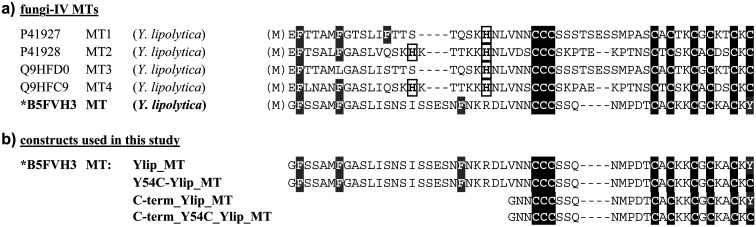
(a) Amino acid sequence alignment of the fungi-IV MT family (cysteine residues are highlighted in black, aromatic amino acid residues in grey, and His residues with a black box). (b) Amino acid sequences of the four constructs investigated here: Ylip_MT, Y54C-Ylip_MT, C-term_Ylip_MT, and C-term_Y54C_Ylip_MT.

This study focuses on a MT sequence from family 11 of fungal-IV MTs, a group of highly homologous sequences identified in the yeast *Yarrowia lipolytica* strain CLIB 122/E 150. Members of this family share a conserved cysteine distribution pattern (Fig. 1). The genes encoding MT1 and MT2 are located in close proximity on chromosome A, whereas the genes for MT3 and MT4 are adjacent on chromosome C (Fig. S1).^33,34^ Transcription of all four genes (MT1–4) is upregulated upon copper exposure.^35^ Genomic sequencing further revealed an additional putative MT gene on chromosome E, designated Ylip_MT in this study (B5FVH3 MT, Fig. 1).

Ylip_MT exhibits several distinctive sequence features that make it an attractive model system for studying Cu(i) coordination. First, it lacks histidine residues, including the otherwise highly conserved His at position 21 (MT1 and 3) or 22 (MT2 and 4), which is replaced by arginine. As a result, cysteine thiolates represent the only potential metal-coordinating ligands. Second, the sequence contains the conserved central CCC motif, raising the question of whether all three cysteine residues within this stretch participate equally in Cu(i) coordination. Third, in contrast to the other family members, Ylip_MT lacks a conserved C-terminal cysteine residue, which is replaced by tyrosine. Finally, the N-terminal region is unusually long and completely devoid of cysteine residues.

These features define three key structural elements whose roles in Cu(i) binding remain unclear: (i) the cysteine-free N-terminal segment, (ii) the central CCC motif, and (iii) the absence of the conserved C-terminal cysteine. In particular, it is unknown whether all cysteine residues contribute equally to Cu(i) coordination or whether specific sequence contexts modulate their reactivity.

To systematically address these questions, we designed a set of targeted variants. To probe the role of the C-terminal region, a Y54C mutant was generated to restore the canonical cysteine residue. To evaluate the contribution of the cysteine-free N-terminus, truncated constructs comprising only the C-terminal domain (C-term_Ylip_MT) were investigated, along with the corresponding Y54C variant (C-term_Y54C_Ylip_MT) (Fig. 1). This design enables us to disentangle the influence of global sequence architecture and local cysteine motifs on metal binding.

Despite these distinctive structural features, the spectroscopic and thermodynamic properties of Cu(i) binding to Ylip_MT and its variants have not yet been characterized, representing a significant gap in our understanding of fungal MTs. Here, we address this gap by combining spectroscopic and potentiometric approaches to elucidate the Cu(i) binding properties and thermodynamic stability of these MT constructs.

## 2. Experimental

### 2.1 Chemicals and solutions

Ylip_MT (≥95% purity), C-term_Ylip_MT (≥95%), and C-term_Y54C_Ylip_MT (≥98%) were purchased from GL Biochem Ltd (Shanghai, P.R. China) and used as received. All buffers and chemicals were ACS grade and purchased from Sigma-Aldrich (Buchs, Switzerland), Merck (Darmstadt, Germany), Chemie Brunschwig (Basel, Switzerland), and Biosolve (Valkenswaard, Netherlands). Chelex® 100 resin was obtained from Bio-Rad (Cressier, Switzerland). Luria–Bertani (LB) media used for *E. coli* expression were from Roth AG (Arlesheim, Switzerland); the isotopically labeled chemicals tris(hydroxymethyl)aminomethane (Tris-D^11^, 98%) and ammonium chloride (^15^N, 99%) were from Cambridge Isotope Laboratories (ReseaChem, Burgdorf, Switzerland) and D_2_O (99.8%) was from Armar Chemicals (Dottingen, Switzerland). Plasmid pRK793 for the production of tobacco etch virus (TEV) protease in *E. coli* was a gift from David Waugh (Addgene plasmid #8827).^36^ All solutions were degassed under vacuum, followed by saturation with nitrogen. When complete absence of oxygen was required, solutions were degassed *via* three to four freeze–thaw cycles at a vacuum line and saturated with argon in the glovebox.

### 2.2 Cloning, expression, and purification of Ylip_MT and Y54C_Ylip_MT

The genomic sequence encoding for Ylip_MT was codon-optimized for expression in *E. coli* using the Codon Optimization Tool from Integrated DNA Technologies (Fig. S2), purchased from Microsynth AG (Balgach, Switzerland) and cloned into the expression vector pGEX-4T-1 using the BamHI and XmaI restriction sites. Subsequently, a point mutation was introduced to obtain the vector with the Y54C_Ylip_MT sequence (Fig. S3). Transformation into chemically competent bacterial *E. coli* XL1Blue cells was achieved by heat shock at 42 °C for 45 seconds in a water bath. The culture was placed on 1.5% agar plates containing 100 μg mL^−1^ ampicillin and was incubated overnight at 37 °C.

The proteins were expressed in LB or TB media containing 0.1 mg mL^−1^ ampicillin. The ^15^N-labeleled peptides for heteronuclear NMR experiments were expressed in M9 minimal media using ^15^N ammonium chloride as the nitrogen source. Overexpression was carried out for 6 h at 30 °C or overnight at 17 °C after induction with 1 mM isopropyl-β-d-thiogalactopyranoside (IPTG) at an OD_600_ value of approximately 0.6. Cell pellets were harvested by centrifugation and lysed by sonication. For initial purification of GST-tagged MTs, first a GST affinity column (GSTPrep FF 16/10, GE Healthcare, Glattbrugg, Switzerland) was used with 1× phosphate-buffered saline (PBS), pH 7.3, as the equilibration and loading buffer and 5 mL of 50 mM reduced glutathione (GSH) in 50 mM Tris-HCl (pH 8) as the elution buffer. Afterwards, samples were dialyzed against TRIS-HCl buffer to remove GSH. The GST tag was cleaved with TEV protease for 3 h at 34 °C in the dialysis buffer (50 mM TRIS-HCl, pH 8) by adding 0.5 mM 1,4-dithiothreitol (DTT) and 0.1 mM ethylenediaminetetraacetic acid (EDTA, pH 8) and using a TEV : peptide ratio of 1 : 30 (w/w). TEV protease was produced according to the standard procedure with some modification.^37,38^ Cleaved MTs were further purified *via* size exclusion chromatography (HiLoad 16/600 Superdex 75 pg, 10 mM ammonium acetate, pH 7.8) and lyophilized overnight. The apo-MTs were resuspended in 10 mM HCl and 100 mM tris(2-carboxyethyl)phosphine (TCEP) and purified with a Superdex Peptide 10/300 GL column under anaerobic conditions (a Coy Lab Vinyl Anaerobic Chamber equipped with a palladium catalyst and filled with a 5% hydrogen/95% nitrogen gas mixture). Protein masses were confirmed using electrospray ionization mass spectrometry (ESI-MS). The final concentrations of the purified proteins were determined by thiol quantification using the 2,2′-dithiopyridine (2-PDS) assay.^39^

### 2.3 Removal of unbound and loosely bound Cu(i) ions with Chelex® 100

A 20 mM Cu(i) stock solution was prepared from tetrakis(acetonitrile)copper(i) tetrafluoroborate ([Cu(MeCN)_4_BF_4_], Sigma Aldrich, Buchs, Switzerland), and 2–3% (v/v) acetonitrile was added to stabilize the Cu(i) oxidation state. Inside the anaerobic chamber, apo-peptides (*c* = 20 µM) were incubated with 6.5 equiv. of Cu(i) and the excess was removed by treatment with a small amount of Chelex® 100 beads at pH 7.4 in 10 mM Tris-HCl, 10 mM NaCl buffer for 20 min. The beads were removed by centrifugation and the amount of Cu(i) bound to the respective peptide was subsequently determined using flame atomic absorption spectroscopy (F-AAS, AA204FS spectrometer, Varian, Zug, Switzerland) by direct dilution of peptides into 0.2 M HNO_3_.

### 2.4 UV titration of MTs with Cu(i)

All titration experiments were performed using a 1 cm septum sealed quartz cuvette (volume 800 µl), which was filled under strictly anaerobic conditions inside the glovebox. The apo-proteins were diluted with water and Tris/NaCl buffer to a final concentration of 10 mM Tris-HCl and 10 mM NaCl, pH 7.4 with peptide concentrations of 8–15 µM. The initial protein concentrations were calculated using the 2-PDS assay, the metal ion solution was added using a Hamilton glass syringe (25 µL) and the total amount of added Cu(i) ions was verified at the end of each titration using F-AAS. All UV spectra were recorded on a Cary 60 UV-vis spectrophotometer (Agilent Technologies, Portmann Instruments AG, Biel-Benken, Switzerland) at 25 °C. To confirm the absence of thiol group oxidation when performing the titrations in a sealed cuvette under a normal oxygen atmosphere, a control spectrum was recorded inside the glovebox (UV5BIO, Mettler Toledo, Greifensee, Switzerland), and it revealed no differences.

### 2.5 CD spectroscopy

All CD spectra were collected in 10 mM Tris-HCl and 10 mM NaCl, pH 7.4, using a 1 cm septum sealed quartz cuvette (volume 800 µL) in a J-715 spectropolarimeter (Jasco, Japan) at 25 °C in the range of 200–600 nm with a scanning speed of 500 nm min^−1^ using three acquisitions per spectrum. Protein concentrations were the same as for the UV titrations. Far-UV CD spectra were obtained in a 0.01 cm quartz cuvette with protein concentrations of 50 µM. Secondary structure contents were analysed using DichroWeb as described in the Results section.

### 2.6 Potentiometric measurements

Potentiometric measurements were performed at a constant temperature of 25 °C under a nitrogen atmosphere using an automated T50 titrator (Mettler Toledo, Greifensee, Switzerland) and a combined micro glass pH electrode (Mettler Toledo DGi101-SC). Stability constants for proton and Cu(i) complexes were calculated from titrations (two titration curves) carried out over the pH range 2–8 using a total volume of 1.3 mL. The glass cell was equipped with a magnetic stirring system, a buret delivery tube (1 mL) and an inlet–outlet tube for nitrogen. Starting solutions contained the respective apo-proteins in a concentration between 0.15 and 0.25 mM in 10 mM HCl at 100 mM KCl ionic strength and were supplemented with 0 to 4 equivalents of Cu(i). The titrations were performed using carbonate-free 0.1 M NaOH, which was purchased from Sigma-Aldrich and then potentiometrically standardized with potassium hydrogen phthalate as a primary standard.^40,41^ Before each measurement, the electrode was calibrated by titrating a reference solution just containing 10 mM HCl with 0.1 M NaOH. The Glee program was used for glass electrode calibration.^42^ Apo-protein concentrations ranged between 0.15 and 0.25 mM. The metal-to-ligand ratios were 1 : 1, 2 : 1, 3 : 1 and 4 : 1. The HYPERQUAD 2013 program was used for the stability constant calculations.^43^ Reported log *β* values refer to the overall equilibria:pCu + qH + rL = Cu_p_H_q_L_r_
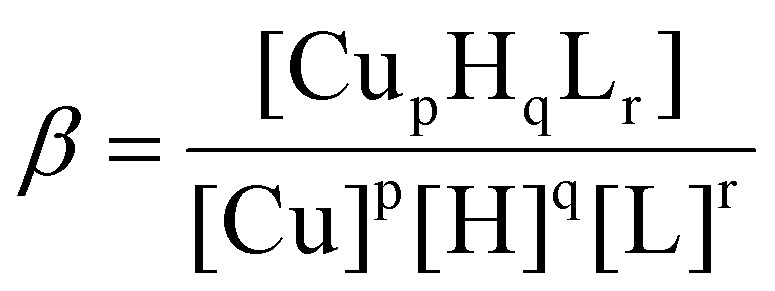
(Charges have been omitted for clarity.)`

Standard deviations were computed using HYPERQUAD 2013 and refer to random errors only. The speciation and competition diagrams were generated using the HySS program.^44^

The details of the calculation of proton and complex formation constants have been described previously.^45^

### 2.7 NMR spectroscopy

The [^1^H–^15^N]-HSQC spectra of Ylip_MT and Y54C_Ylip_MT in their apo-forms and in the presence of 4 equiv. of Cu(i) were recorded using a Bruker Advance™ 600 MHz spectrometer, equipped with a TCI cryoprobe, at a constant temperature of 300 K. For each sample, the total volume was 220 µL and the final protein concentration was 0.15–0.2 mM in 20 mM ^11^D-Tris (50 mM NaCl, pH 8) and 10% D_2_O. All samples were prepared under anaerobic conditions, filled into 5 mm symmetrical micro-NMR tubes (Shigemi Co., Ltd, Japan) and sealed with parafilm.

### 2.8 Dynamic light scattering (DLS) measurements

DLS measurements were performed using a DynaPro Titan instrument equipped with a temperature controlled micro sampler (Wyatt Technology Corporation, Santa Barbara, CA, USA). The measurements were performed using 50 µL of 100 µM samples at 20 °C in a buffer containing 10 mM Tris-HCl and 10 mM NaCl, pH 7.4, with 10 data acquisitions per measurement. Each sample was recorded at least five times and evaluated using the Dynamics® software (Wyatt Technology Corporation). Before each measurement, samples were incubated with exactly 4 equiv. of Cu(i) and centrifuged at 13 200 rpm for 30 min under anaerobic conditions.

### 2.9 MS spectrometry

For ESI-MS measurements, the samples were prepared in 10 mM ammonium formate buffer at pH 4.5. The respective apo-protein was loaded with seven equivalents of Cu(i), incubated for 20 min with Chelex® 100 to remove the unbound or unspecifically coordinated Cu(i) ions, and separated from the resin by centrifugation. Prior to the measurements, the metal–protein complexes were desalted using a C_18_ ZipTip (Millipore, Billerica, MA, USA). The solutions were infused through a fused silica capillary (ID 75 μm) at a flow rate of 1 µL min^−1^ and sprayed through a PicoTips (ID 30 μm). The latter were obtained from New Objective (Woburn, MA). Nano ESI-MS analyses of the samples were performed with a Synapt G2_Si mass spectrometer, and the data were recorded with the MassLynx 4.2 software (both Waters, UK). Mass spectra were acquired in the positive-ion mode by scanning an *m*/*z* range from 400 to 4000 Da with a scan duration of 1 s and an interscan delay of 0.1 s. The spray voltage was set to 2.7 kV, the cone voltage to 50 V, and the source temperature to 80 °C. The recorded *m*/*z* data were then deconvoluted into mass spectra (monoisotopic masses) by applying the maximum entropy algorithm MaxEnt1 (MaxLynx) with an output mass resolution of 0.03 Da per channel and the Uniform Gaussian Damage Model at the half height of 0.1 Da.

For MALDI-TOF MS, the Cu(i)-loaded protein was prepared in 50 mM ammonium acetate buffer at pH 4.5. A double-layer spotting technique was applied, whereby 0.2 μL of matrix solution consisting of saturated sinapic acid (SA) in EtOH was first deposited onto a ground steel MALDI target plate (Bruker) and allowed to dry completely. Then, 1 μL of the protein sample was mixed with 1 μL of SA matrix solution (saturated in H_2_O/MeCN at 2 : 1 containing 0.1% TFA). From this mixture, 0.5 μL was drop-cast onto the previously dried matrix layer and allowed to dry prior to analysis. MALDI-TOF MS experiments were performed using an Autoflex Speed time-of-flight mass spectrometer (Bruker Daltonics, Bremen, Germany) equipped with a Bruker Smartbeam™-II laser operating at a wavelength of 355 nm in positive linear mode. Instrument control and data acquisition were carried out using the Bruker Daltonics flexControl software (version 3.4). Spectra were accumulated over 10 000–15 000 laser shots and recorded between *m*/*z* 1000 and 20 000. Average masses were calibrated using signals from a protein mixture consisting of insulin ([M + H]^+^_avg_ = *m*/*z* 5734.51 Da), cytochrome C ([M + 2H]^2+^_avg_ = *m*/*z* 6180.99 Da), myoglobin ([M + 2H]^2+^_avg_ = *m*/*z* 8476.65 Da), ubiquitin I ([M + H]^+^_avg_ = *m*/*z* 8565.76 Da), cytochrome C ([M + H]^+^_avg_ = *m*/*z* 12 360.97 Da), and myoglobin ([M + H]^+^_avg_ = *m*/*z* 16 952.30 Da), all obtained from the Bruker Protein Calibration Standard I mixture (mass range *ca.* 4000–20 000 Da).

## 3. Results and discussion

### 3.1 Cu(i) binding capacity of the constructs

The maximum Cu(i) binding capacities of the four constructs were determined using flame atomic absorption spectroscopy (F-AAS) in combination with the PDS assay, which quantifies protein concentration by detecting cysteine thiolate groups.^39^ The results consistently indicated coordination of approximately four Cu(i) ions per MT molecule:

Ylip_MT4.1 ± 0.1

Y54C_Ylip_MT 4.43 ± 0.03

C-term-Ylip_MT 3.98 ± 0.08

C-term-Y54C_Ylip_MT 3.95 ± 0.03

Additionally, matrix-assisted laser desorption/ionization time-of-flight mass spectrometry (MALDI-TOF MS) of Cu(i)-loaded Ylip_MT and electrospray ionization mass spectrometry (ESI-MS) of C-term-Ylip_MT and C-term-Y54C_Ylip_MT predominantly show the Cu_4_MT species as the main form (Fig. S4), further corroborating the stoichiometry determined by F-AAS. No ESI-MS spectrum was obtained for Y54C_Ylip_MT, potentially due to issues with ionization or sample stability during analysis. The binding capacities of the different Ylip_MT constructs are thus slightly lower compared to those of other small fungal MTs (*e.g.*, from *A. bisporus* and *N. crassa*)^26,27^ but comparable to that of the Cu_4_Cys_9_ cluster observed in human MT3.^28,30,31^ These findings confirm that the N-terminal cysteine-free segment does not affect the maximum Cu(i) binding capacity and that the shorter constructs can serve as a robust model for studying metal ion coordination.

### 3.2 Spectroscopic investigations of Cu(i) binding to Ylip_MT and Y54C_Ylip_MT

To assess Cu(i) coordination to fungal Ylip_MT, Y54C_Ylip_MT, and their truncated constructs, UV-vis and circular dichroism (CD) spectroscopy were employed.

#### 3.2.1 Analysis with UV spectroscopy

**Fig. 2:**
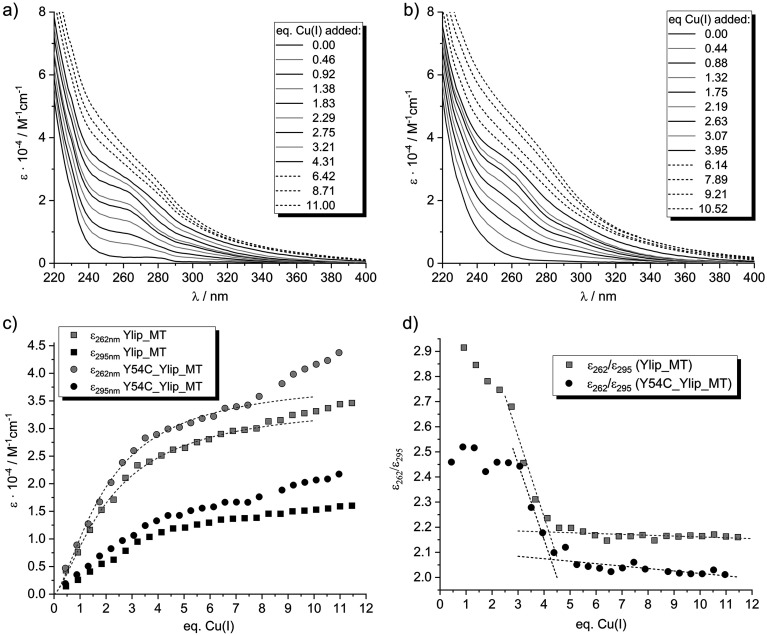
Titration of Ylip_MT and Y54C_Ylip_MT with Cu(i) monitored using UV spectroscopy. (a) Selected UV spectra for Ylip_MT and (b) selected UV spectra for Y54C_Ylip_MT. (c) Plots of molar absorptivity values at 262 nm and 295 nm for both proteins, including data fitting with the Hill equation for the *ε*_262_ data (dotted lines, Table S1). (d) *ε*_262_/*ε*_295_ ratio for both proteins, including linear fits of data to estimate the maximum Cu(i) binding capacity (protein concentrations of 8–15 µM in 10 mM Tris-HCl 7.4 and 10 mM NaCl).

Spectrophotometric titrations were performed by stepwise addition of Cu(i) to the apo-proteins. Upon Cu(i) binding, the UV-vis spectra developed a broad absorption envelope superimposed on the intrinsic protein backbone transitions, with distinct shoulders emerging at approximately 262 and 295 nm (Fig. 2a, b and S5a, b). The prominent shoulder at 262 nm is attributed to sulfur-to-Cu(i) charge-transfer (LMCT) transitions, whereas the weaker shoulder at 295 nm arises from cluster-centered transitions associated with d^10^–d^10^ interactions in Cu(i) clusters containing short Cu(i)–Cu(i) distances.^46,47^ For all constructs, the intensity of the LMCT band at 262 nm increases roughly linearly up to ∼3.0 equivalents of Cu(i) (Fig. 2c and S5c). Beyond this point, additional Cu(i) results in a more gradual increase without clear saturation. The absorptivity at 295 nm likewise increases nearly linearly up to ∼4.0 equivalents, after which the slope decreases.

To better differentiate between LMCT contributions and cluster-centered transitions, the ratio *ε*_262_/*ε*_295_ was plotted as a function of added Cu(i) (Fig. 2d and S5d). This allows two important conclusions. First, the evolution of the ratio argues against strictly cooperative binding in which only a single dominant species forms. In a purely cooperative scenario, one would expect the spectral shape to remain constant during titration, with increasing Cu(i) leading only to a proportional increase in intensity. Under such conditions, the ratio of absorptivities at two selected wavelengths would remain constant throughout the titration. Second, in contrast to this expectation, the *ε*_262_/*ε*_295_ ratio shows changes up to approximately four equivalents of Cu(i), becoming constant only thereafter. This clearly indicates that the nature of the Cu(i) coordination environment evolves during titration. Notably, for all constructs, the ratio decreases markedly between three and four equivalents of Cu(i), reflecting a disproportionate increase in the 295 nm band. This behaviour is characteristic of Cu(i)–thiolate cluster formation, in which additional bridging thiolate ligands promote Cu(i)–Cu(i) interactions and stabilize multinuclear assemblies.^46–49^ At Cu(i) loadings above ∼4 equivalents, the ratio reaches a plateau. This behaviour likely reflects the onset of uniform scattering contributions, possibly due to minor Cu(i) precipitation, affecting the measured wavelength range similarly at both wavelengths.

Interestingly, differences between constructs are evident. For the Y54C mutant proteins, the *ε*_262_/*ε*_295_ ratio remains nearly constant during the initial titration phase (up to ∼3 equivalents), whereas both full-length and truncated wild-type sequences exhibit a gradual decrease over this range. The comparatively stronger early increase in cluster-centered transitions in the wild-type proteins suggests that some bridging cysteine residues may already participate in metal coordination during binding of the first three Cu(i) equivalents, possibly reflecting the reduced number of cysteine ligands available.

In summary, the UV data support a two-stage model of Cu(i) coordination. In the first stage, up to ∼3 equivalents, Cu(i) ions bind primarily to terminal thiolate ligands, resulting predominantly in LMCT transitions. In the second stage, completed at approximately four equivalents, recruitment of bridging thiolates leads to formation of multinuclear Cu(i)–thiolate clusters, reflected by the pronounced increase in absorptivity at 295 nm. The close similarity in spectral behaviour between the full-length proteins and their truncated counterparts further indicates that the cysteine-free N-terminal region of Ylip_MT does not directly participate in Cu(i) coordination.

#### 3.2.2 Analysis with CD spectroscopy

**Fig. 3:**
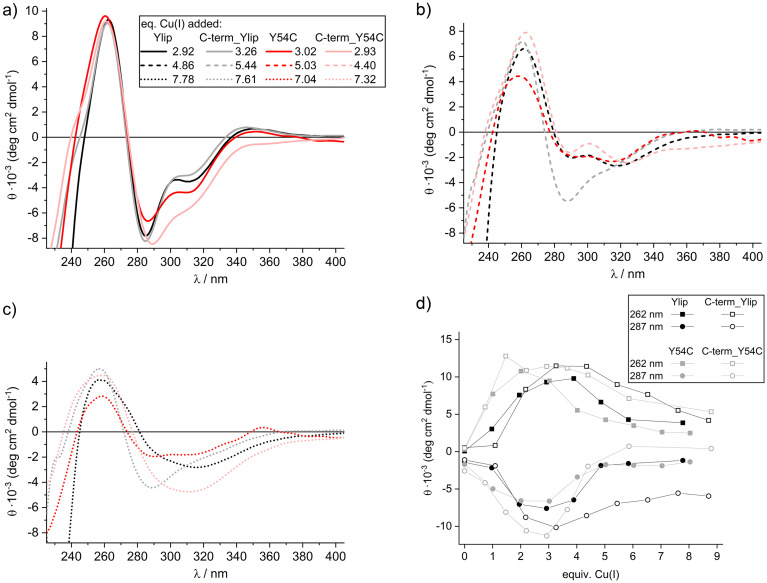
Titration of Ylip_MT, Y54C_Ylip_MT, and the C-terminal truncated constructs with Cu(i) monitored with CD spectroscopy. Protein concentrations of 8–15 µM in 10 mM Tris-HCl 7.4 and 10 mM NaCl. Overlay of spectra upon addition of approximately (a) 3, (b) 4.5–5.5, and (c) 7–8 equiv. of Cu(i) (for precise amounts, see the legend in a). The spectra of the truncated constructs show roughly 20% higher ellipticity values (see part d) and were normalized on the 262 nm values of the full-length constructs for better comparison. (d) Plots of molar ellipticity values at 262 nm and 287 nm for all constructs (see also S6 and S7). No normalisation of values was performed.

To assess structural changes associated with Cu(i) binding, we also monitored the titrations using CD spectroscopy,^28,49–51^ and the results further corroborate our conclusions drawn from the UV spectra. The addition of up to roughly three equivalents of Cu(i) resulted in the development of defined CD spectra, with positive ellipticity observed at 262 and 345 nm and negative ellipticity at 287 and 315 nm (Fig. 3a, S6, and S7). As Cu(i) was further added beyond saturation, the ellipticity values gradually declined (Fig. 3d), and the distinct bands between 280 and 320 nm lost their specific profiles, suggesting a partial unfolding or structural rearrangement (Fig. 3b and c). The CD spectra for Ylip_MT and C-term-Ylip_MT were very similar throughout the titration. In both cases, ellipticity at 262 nm peaked upon addition of 3–4 equivalents of Cu(i), with a corresponding minimum at 287 nm for Cu(i) equivalents between 2 and 4. The notable difference was that C-term_Ylip_MT maintained significant negative ellipticity at 287 nm even at higher Cu(i) levels, whereas the other constructs exhibited only a broad band under these conditions. These results indicate that, by 3 equivalents of Cu(i), most of the cysteine thiolate groups are engaged in binding, with only minimal additional binding occurring upon addition of the fourth equivalent. Cu(i) additions above the saturation point of 4 equivalents seem to lead to the formation of less defined structures.

The titrations of the two Y54C mutants yielded comparable spectra with subtle differences. For instance, the band at 315 nm was more pronounced at sub-saturation Cu(i) concentrations compared to the wild-type constructs, and in C-term_Y54C_Ylip_MT the negative band at 287 nm was slightly shifted to 289 nm. Moreover, maximum and minimum ellipticity values at 262 and 287 nm were reached at lower Cu(i) equivalents (2 and 2–3 for Y54C_Ylip_MT, and 2–3 and 3 for C-term_Y54C_Ylip_MT), suggesting that the additional C-terminal cysteine in these mutants reduces steric strain and facilitates earlier binding of all cysteine thiolates.

Finally, the comparable extrema observed during Cu(i) titrations of the two plant MTs, γ-E_c_-1 and cicMT2, of the two domains of mouse MT1 and human MT3, as well as for the Cu(i)-thionein from *S. cerevisiae* in the UV-vis region (262 nm, 285–295 nm, and 300–400 nm) further corroborate these spectral features as characteristic signatures of Cu(i)–thiolate cluster formation.^28,48–50,52^

#### 3.2.3 Insights into the metalation pathway

To integrate the spectroscopic observations with the stepwise binding model derived from potentiometry (*vide infra*), we estimated how many cysteine thiolates participate in Cu(i) coordination at each titration step. The intensity of the LMCT band at 262 nm (Fig. 2 and S5) provides a useful proxy, as it directly reflects sulfur-to-Cu(i) charge transfer and thus correlates with the number of coordinated thiolates. As a first approximation, we determined the maximum absorptivity at 262 nm after the addition of four equivalents of Cu(i), corresponding to full metal loading. Assuming that all cysteine residues contribute equally to the LMCT signal, a necessary but simplified assumption, this total absorptivity was divided by eight for the wild-type constructs and by nine for the Y54C variants. The resulting average per-cysteine absorptivities were 3088, 3379, 3189, and 3784 M^−1^ cm^−1^ for Ylip_MT, C-term_Ylip_MT, Y54C_Ylip_MT, and C-term_Y54C_Ylip_MT, respectively (Tables S2 and S3). These values are consistent with the literature data. For example, human Cu_4_-MT3 exhibits an *ε*_max_ of ∼31 000 M^−1^ cm^−1^ at 262 nm, with 8–9 thiolate ligands contributing to cluster formation.^47^ Our estimates are therefore in good agreement with a Cu_4_Cys_8–9_ cluster model.

**Fig. 4:**
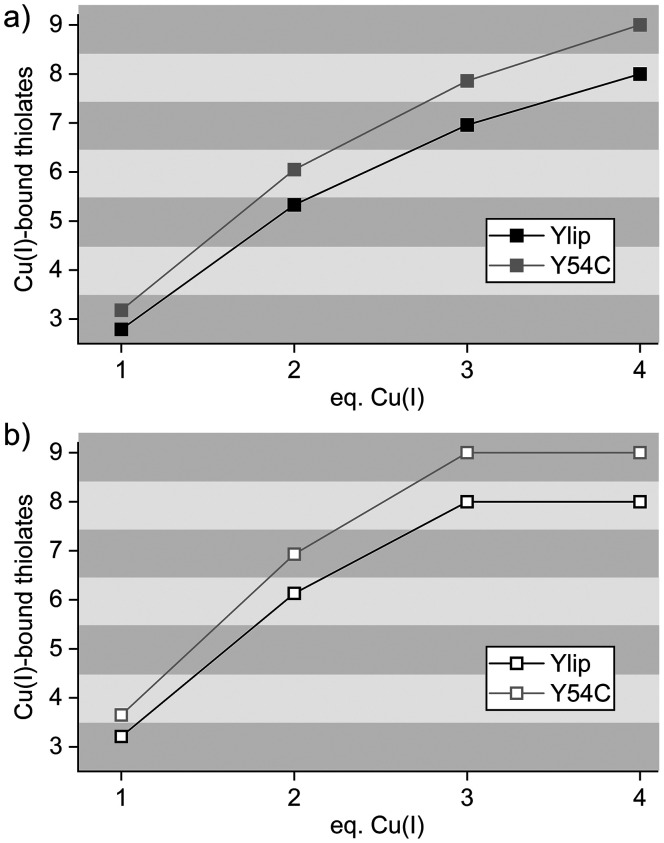
Estimated number of Cu(i)-coordinating thiolate groups in Ylip_MT and Y54C_Ylip_MT derived from UV absorption data (see Fig. 2, Tables S1–S3). (a) Calculation assuming that full thiolate participation is reached after the addition of 4 equivalents of Cu(i). (b) Calculation assuming that all thiolate groups are fully engaged by 3 equivalents of Cu(i). The plots are shown with a rastered background to facilitate visualization of the number of coordinating thiolates.

To reduce experimental uncertainty, we used absorptivity values obtained from a curve fit of the titration data rather than relying solely on raw measurements (Fig. 2 and S5, Table S1). The resulting analysis for the two full-length constructs is shown in Fig. 4a (the truncated variants behave nearly identically and are therefore not shown). According to this model, binding of the first Cu(i) equivalent involves approximately three cysteine thiolates in both constructs, consistent with the typical trigonal-planar coordination geometry of Cu(i) in MTs.^25^ Addition of the second and third equivalents progressively increases the number of participating thiolates, reaching approximately seven (wild type) and eight (Y54C mutant) after three equivalents. Full saturation corresponds to engagement of all eight or nine cysteines, respectively.

An alternative scenario must also be considered. It is conceivable that most cysteine thiolates are already engaged after addition of three equivalents of Cu(i), and that the changes observed upon further metal addition primarily reflect structural rearrangements rather than recruitment of new ligands. Several observations support this possibility:

(i) the UV-vis data indicate that cluster formation becomes significant once more than three equivalents of Cu(i) are added (Fig. 2d),

(ii) the CD spectra reach maximum ellipticity at approximately three equivalents or earlier (Fig. 3d), and

(iii) the increase in *ε*_262_ may not exclusively report on the number of terminal thiolates but may also intensify when terminal ligands convert into μ-bridging coordination modes during cluster assembly. Under this scenario, the additional increase in absorptivity between three and four equivalents would primarily reflect formation of bridging thiolates rather than recruitment of additional cysteine residues for binding.

The calculation based on this alternative scenario is shown in Fig. 4b and results in approximately one additional coordinating thiolate per titration step compared to the first model.

### 3.3 Potentiometric studies of Cu(i) binding to Ylip_MT, C-term_Ylip_MT, and C-term_Y54C_Ylip_MT

To further elucidate the Cu(i) metalation process, we performed potentiometric pH titrations, which monitor protonation equilibria over a broad pH range and thereby probe the coupling between proton release and Cu(i) coordination. Although potentiometry does not directly determine metal stoichiometry, it sensitively detects proton dissociation events from ionizable groups involved in metal binding. Because coordination of Cu(i) to cysteine thiolates is coupled to thiol deprotonation, metal binding induces measurable shifts in the apparent p*K*_a_ values of these groups. By comparing the p*K*_a_ values in the absence and presence of increasing Cu(i) equivalents, individual proton dissociation events can be assigned to successive metal-binding steps. In this way, the method resolves the metalation process as a sequence of stepwise equilibria across the entire accessible pH range. Fitting the titration data (Fig. S8) to appropriate equilibrium models yields stepwise stability constants for Cu(i) binding. Potentiometry thus provides thermodynamic insight into the progressive engagement of ionizable ligands during metal coordination, complementing the information obtained from spectroscopic methods.

The full-length proteins Ylip_MT and Y54C_Ylip_MT contain 19 (5 Lys, 8 Cys, 2 Asp, 1 Glu, and 1 Arg, plus the C- and N-termini) and 20 protonation sites (plus an extra Cys), respectively. This high number of titratable groups renders the potentiometric analysis complex. Since our spectroscopic data indicate that the cysteine-free N-terminal region does not significantly contribute to Cu(i) binding, we first focused on the corresponding C-terminal fragments. These truncated constructs contain fewer protonation sites, 15 for C-term_Ylip_MT (4 Lys, 8 Cys, and 1 Asp, plus the C- and N-termini) and 16 for C-term_Y54C_Ylip_MT (plus one additional Cys), thereby simplifying the system.

All titrations were initiated from the apo-proteins dissolved in 10 mM HCl (pH 2). Based on previous results showing a Cu(i) binding capacity of four equivalents for both Ylip_MT and its Y54C mutant, titrations were performed at five protein-to-metal ratios (1 : 0, 1 : 1, 1 : 2, 1 : 3, and 1 : 4). Although data were collected over a pH range of 2–11, only values up to pH 8–9 were included in the calculations to avoid complications from oxidative processes at higher pH. Within this evaluated pH range stepwise deprotonation of 11 sites was observed for both truncated constructs. For C-term_Ylip_MT, these correspond to the N- and C-termini, eight cysteine residues, and one aspartate. For C-term_Y54C_Ylip_MT, the deprotonating groups comprise the C-terminus, nine cysteines (or eight cysteines plus the N-terminus), and one aspartate. The most deprotonated species are defined as H_4_L for C-term_Ylip_MT and H_5_L for C-term_Y54C_Ylip_MT, while the fully protonated peptide species are denoted (H_4_L)H_11_ and (H_5_L)H_11_, respectively (Table 1). Accurate determination of individual p*K*_a_ values in the alkaline region is challenging because the N-terminal –NH_3_^+^ group and the most basic cysteine residues deprotonate within a narrow pH window (8–9). In C-term_Y54C_Ylip_MT, the highest p*K*_a_ (assigned to either the N-terminal–NH_3_^+^ group or the most alkaline cysteine) exceeds 9.3 (Table 1) and could therefore not be reliably determined under the applied experimental conditions.

**Table 1:** Protonation constants of apo-C-term_Ylip_MT and apo-C-term_Y54C_Ylip_MT determined by potentiometric pH titration (initial solution: 10 mM HCl) at *I* = 0.1 M KCl, *T* = 298 K. Standard deviations are given in parentheses as uncertainties on the last significant digit

C-term_Ylip_MT				C-term_Y54C_Ylip_MT		
Species	log *β*	p*K*_a_	Protonation site	p*K*_a_	log *β*	Species
(H_4_L)H_11_	75.3(1)	3.5	C-terminus	2.7	76.6(1)	(H_5_L)H_11_
(H_4_L)H_10_	71.8(1)	3.5	Asp-COOH	2.8	73.9(1)	(H_5_L)H_10_
(H_4_L)H_9_	68.3(1)	4.3	Cys-SH	4.2	71.7(1)	(H_5_L)H_9_
(H_4_L)H_8_	64.0(1)	6.8	Cys-SH	6.8	66.9(1)	(H_5_L)H_8_
(H_4_L)H_7_	57.2(1)	7.5	Cys-SH	7.8	60.1(1)	(H_5_L)H_7_
(H_4_L)H_6_	49.7(1)	7.7	Cys-SH	7.8	52.3(1)	(H_5_L)H_6_
(H_4_L)H_5_	42.0(1)	8.0	Cys-SH	8.5	44.5(1)	(H_5_L)H_5_
(H_4_L)H_4_	34.0(1)	8.3	Cys-SH	8.5	36.0(1)	(H_5_L)H_4_
(H_4_L)H_3_	25.7(1)	8.5	Cys-SH	9.1	27.5(1)	(H_5_L)H_3_
(H_4_L)H_2_	17.2(1)	8.6	Cys-SH	9.1	18.4(1)	(H_5_L)H_2_
(H_4_L)H_1_	8.6(1)	8.6	N-terminus/Cys-SH^a^	9.3	9.3(1)	(H_5_L)H_1_

a N-terminus for C-term_Ylip, N-terminus or Cys-SH for C-term_Y54C_Ylip_MT.

#### 3.3.1 Protonation constants of apo-C-term-Ylip_MT and apo-C-term-Y54C_Ylip_MT

Table 1 presents the protonation constants (log *β*) and corresponding p*K*_a_ values for apo-C-term-Ylip_MT and apo-C-term-Y54C_Ylip_MT.

The two lowest proton dissociation constants – 3.5 for apo-C-term-Ylip_MT and 2.7/2.8 for apo-C-term-Y54C_Ylip_MT – are assigned to the carboxylic acid groups of the C-terminus and the aspartic acid side chain. These values are consistent with the literature data of C-termini and carboxylic groups in folded proteins.^53^ For C-term-Ylip_MT, nine additional protonation constants were determined that are consequently assigned to the eight cysteine residues and the N-terminal amino group. While for C-term-Y54C_Ylip_MT ten additional protonation constants were expected in the covered pH range, only nine were obtained. These correspond either to all nine cysteine residues or to eight cysteines plus the N-terminal-NH_3_^+^ group. A reliable differentiation between these possibilities is not feasible due to their overlapping deprotonation ranges. The p*K*_a_ values for the four lysine residues in both MTs – as well as the remaining undetected group in C-term-Y54C_Ylip_MT (either the additional cysteine or the N-terminus) – are expected to exceed 9.5–10 and therefore lie outside the pH window evaluated here.

The p*K*_a_ values of cysteine thiolate and N-terminal-NH_3_^+^ groups are usually found in a range of 8–9.^53–57^ However, deviations from this range have been reported, particularly in enzymes containing redox-active cysteine residues such as the thiol disulfide oxidoreductase DsbA.^53,58^ Lowered p*K*_a_ values may result from hydrogen bonding to other residues or by surrounding positive charges of other side chains as shown for, *e.g.*, a protein from the thioredoxin-fold family.^59^ More recently, we observed a pronounced downward shifted p*K*_a_ also in the apo-form of a MT. Using potentiometric pH titrations similar to the study performed here, a p*K*_a_ value of 4.4 was determined for one cysteine thiol group in an eight cysteine residues containing small MT from the aquatic fungus *Heliscus lugdunensis*, while the other seven cysteines had p*K*_a_ values >6.6.^45^ Similarly, a p*K*_a_ value of 4.7 was recently assigned to a thiol in the apo-form of a small cysteine-rich motif from the CopY repressor, again using potentiometry.^60^ These findings clearly demonstrate that the p*K*_a_ range of cysteine residues in metal-binding proteins can be considerably broader than commonly assumed.

Notably, also in the constructs investigated here, one residue in each case exhibits a markedly lower p*K*_a_ value (4.3 for C-term-Ylip_MT and 4.2 for C-term-Y54C_Ylip_MT). Consistent with previous observations and by exclusion of other ionizable groups, this low p*K*_a_ can only be attributed to a cysteine thiol group. A plausible explanation is strong hydrogen-bond stabilization of the thiolate, which would significantly lower its p*K*_a_. Potential hydrogen-bond donors include one of the lysine side chains or a neighboring, still protonated cysteine residue.^58,61^ The reason why such a pronounced effect is observed for only one single thiol among many remains unclear. Interestingly, however, a comparable phenomenon has been reported for polyhistidine-tagged peptides, where one histidine residue displays a substantially lower p*K*_a_ (*e.g.*, 4.70 for the DHDHDHHHHHHPGSSV-NH_2_ peptide (N-DpH)) relative to the remaining histidines (5.40 to 7.51).^62^

#### 3.3.2 Addition of Cu(i) – changes in protonation constants in C-term_Ylip_MT and C-term_Y54C_Ylip_MT

Tables S5 and S6 present the stepwise complex formation constants and calculated p*K*_a_ values for the C-terminal fragments of Ylip_MT and Y54C_Ylip_MT when the apo-proteins were titrated with base in the presence of Cu(i). Fig. 6 presents these calculated p*K*_a_ values, while species distribution diagrams for the various MT : Cu(i) ratios (from 1 : 1 to 1 : 4) are shown in Fig. S9 and S10. These diagrams illustrate the pH-dependent abundance of the different protonation states.

For several protonation states, individual stepwise dissociation constants were not resolved because two or more protons were released within a very narrow pH range. In these cases, the reported p*K*_a_ values are the logarithm of an average one-proton dissociation constant. Upon Cu(i) binding, the p*K*_a_ values of individual cysteine thiol groups decrease relative to the values of the apo form. Previous studies have reported that coordination of Cd(ii) and Zn(ii) to metallothioneins decreases the apparent p*K*_a_ of thiols to roughly 3.5 and 4.5, respectively.^45,63,64^ Given the stronger Cu(i)–Cys interaction, even lower p*K*_a_ values are expected. One of the sparse examples is the apparent p*K*_a_ of 3.1 for a cysteine thiol group in the abovementioned small cysteine-rich motif from the CopY repressor in the presence of one Cu(i) equivalent.^60^

Cu(i)–thiolate complexes in MTs typically adopt digonal or trigonal coordination geometries.^25,65^ Therefore, stepwise coordination of the first Cu(i) equivalent is expected to involve two or three cysteine residues and to lower their p*K*_a_ values accordingly. The first p*K*_a_ values that were fitted from the titration data are 3.1 for the Cu(H_4_L)H_7_ species of C-term_Ylip_MT and 3.2 for the Cu(H_5_L)H_7_ species of C-term_Y54C_Ylip_MT. Accordingly, two cysteine thiol groups each have p*K*_a_ values that are even lower, *i.e.* below ∼3. That very low p*K*_a_ values cannot be determined precisely is commonly observed in complex-formation studies in cases where multiple species coexist within a narrow pH range. Taken together, we can safely conclude that these three thiolates with significantly lowered p*K*_a_ values in each construct are directly involved in Cu(i) binding.

**Fig. 5:**
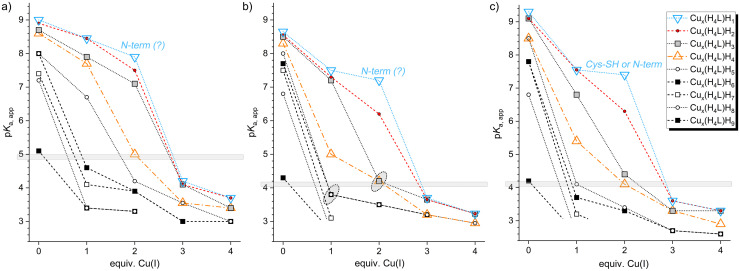
Plots of apparent p*K*_a_ values in the presence of increasing amounts of Cu(i) ions for (a) Ylip_MT (Table S7), (b) C-term_Ylip_MT (Table S5), and (c) C-term_Y54C_Ylip_MT (Table S6). The vertical grey bar marks an arbitrary threshold for Cu(i) coordination, set just below the lowest cysteine thiolate p*K*_a_ value in the respective apo species. The two grey ellipsoids in (b) mark the average of two nearby p*K*_a_ values, which may correspond to groups just above and below this threshold.

Nevertheless, additional protonation sites also display somewhat lower p*K*_a_ values. For C-term_Ylip_MT, an average p*K*_a_ value of 3.8 is obtained for the Cu(H_4_L)H_6_ and Cu(H_4_L)H_5_ species. We may assign one p*K*_a_ to a coordinating thiolate or a thiolate that encounters proximity effects in the folding polypeptide. These may include intramolecular hydrogen bonding or electrostatic stabilization by nearby positively charged residues (*e.g.*, lysine side chains) or coordinated Cu(i) ions. In addition, the CCC motif may further depress neighboring cysteine p*K*_a_ values through proximity effects of adjacent, still protonated thiols.^53^ The second p*K*_a_ value of 3.8 may also still originate from the low cysteine p*K*_a_ seen in the apo-form. In this way we can align the results with the absorption data, *i.e.* 3–4 coordinating cysteine thiolate groups upon addition of one Cu(i) equivalent (Fig. 5). The p*K*_a_ values of the Cu(H_4_L)H_4_, Cu(H_4_L)H_3_, and Cu(H_4_L)H_2_ species range from 5.0 to 7.3 and likely correspond to cysteine residues not directly involved in Cu(i) coordination. The final p*K*_a_ of 7.5 for the Cu(H_4_L)H is attributed to deprotonation of the N-terminal amine, which also does not participate in metal binding and therefore remains close to its free-ligand value. A similar pattern is observed for C-term_Y54C_Ylip_MT. The Cu(H_5_L)H_6_ and Cu(H_5_L)H_5_ species have low values as well (3.7 and 4.1), and the non-coordinating cysteine residues and the N-terminal amine exhibit p*K*_a_ values between 5.4 and 7.6.

At a 2 : 1 metal-to-peptide ratio, five cysteine residues display markedly lowered p*K*_a_ values (≤3.5; 3.4 for the mutant) and hence are clearly directly involved in the coordination of Cu(i) at low pH. The next two cysteines exhibit slightly higher values (4.2 for C-term_Ylip_MT and 4.1 and 4.4 for C-term_Y54C_Ylip_MT) and are thus in the range discussed for thiolates either coordinating or in close proximity to coordinated Cu(i) ions. That results in a maximum number of 6–7 coordinating thiolate groups. The remaining cysteines, not involved in coordination, retain higher p*K*_a_ values (>6.2 and 6.3). Comparison with the number of coordinating thiolates estimated from UV data (Fig. 4 and Table S3), and assuming full thiolate engagement at three Cu(i) equivalents, suggests that two Cu(i) ions are coordinated by six thiolate ligands in the wild-type fragment and seven in the mutant, hence numbers that align with the potentiometric data.

In the presence of three equivalents of Cu(i), six residues in C-term-Ylip_MT display p*K*_a_ values ≤3.2, and seven have values ≤3.3 in C-term_Y54C_Ylip_MT, while also the p*K*_a_ values for the last observed deprotonation step remain relatively low: 3.65 (average) and 3.7 for C-term_Ylip_MT and 3.6 (average) for C-term_Y54C_Ylip_MT. This indicates that, in C-term_Ylip_MT, all eight cysteine thiolates as well as the N-terminal amine group are deprotonated in the presence of three equivalents of Cu(i). On one hand, this agrees well with the UV data. On the other hand, it clearly demonstrates the unexpected – and to our knowledge unprecedented – coordination of Cu(i) by the N-terminal amine. For C-term_Y54C_Ylip_MT, nine sites are deprotonated at low pH, suggesting that either all nine cysteine thiolates, or eight thiolates plus the N-terminus, are involved in Cu(i) binding. However, based on the UV results, it is most likely that all nine thiolate groups are coordinated.

No major changes are observed when evaluating the 4 : 1 metal-to-peptide ratio, except that all p*K*_a_ values further decreased, strongly indicating Cu(i) coordination (≤3.23 for C-term_Ylip_MT and ≤3.3 for C-term_Y54C_Ylip_MT).

#### 3.3.3 Protonation constants of full-length Ylip_MT upon Cu(i) addition

To further evaluate whether the N-terminal amine participates in Cu(i) coordination, we also performed potentiometric titrations on full-length Ylip_MT. Overall, the trends resemble those observed for the truncated constructs, although several differences are noteworthy (Table S7). Most prominently, the lowest p*K*_a_ value assigned to a cysteine residue in the apo form is approximately one unit higher in the full-length protein than in the truncated fragment (5.1 *vs.* 4.3). Accordingly, the threshold used to identify Cu(i)-coordinating thiolates was adjusted (Fig. 5a). Upon Cu(i) addition, the p*K*_a_ values decrease in a pattern similar to that of the truncated construct and is also fully consistent with the UV-derived estimates of coordinating thiolates. Overall, the observed p*K*_a_ shifts are slightly lower than in the truncated constructs. The additional N-terminal sequence thus seems to influence the protonation behaviour, be it *via* shielding of the metal-binding core or by additional non-covalent interactions. At higher Cu(i) loadings (3 : 1 and 4 : 1 metal-to-peptide ratios), the flexible N-terminus of the full-length protein becomes deprotonated as well and may participate in metal ion binding despite its larger distance from the metal–thiolate cluster in the amino acid sequence.

#### 3.3.4 Comparison of stability

**Fig. 6:**
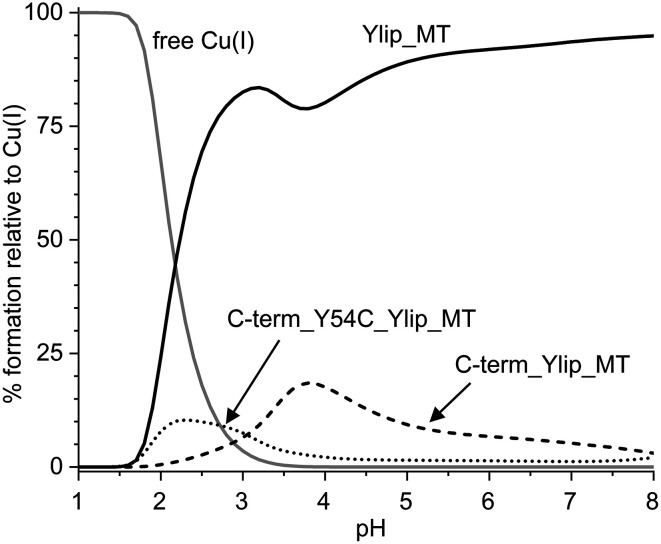
Competition plots for Cu_4_Ylip_MT, C-term_Cu_4_Ylip_MT, and C-term_Cu_4_Y54C_Ylip_MT based on the calculated formation constants (Tables S6–S8) and using equimolar protein concentrations in the presence of four equivalents of Cu(i).

To further understand and compare the Cu(i) binding affinities of full-length Ylip_MT and the two C-terminal fragments obtained from the stepwise deprotonation constants, competition plots were constructed based on the calculated formation constants, using equimolar ligand concentrations in the presence of four equivalents of Cu(i) (Fig. 6). Across the entire pH range, the Cu_4_Ylip_MT complex exhibits the highest stability, suggesting that the long, cysteine-free N-terminal segment in the full-length protein enhances Cu(i) binding and stabilizes complex formation. The complex stabilities of both C-terminal fragments are comparable overall; however, the Y54C mutant exhibits higher Cu(i) binding efficiency below pH 3.5, whereas the wild-type fragment becomes more stable at higher pH values. Given the absence of other ionizable side chains such as histidine within this pH range, protein denaturation is unlikely.^66^ Therefore, the differences in stability constants and apparent p*K*_a_ values of the cysteine thiolates, combined with the presence of an additional thiolate ligand in the mutant, likely contribute to the enhanced Cu(i) binding of the truncated mutant at acidic pH. In contrast, at pH values above 4, the C-term_Cu_4_Ylip_MT complex becomes thermodynamically more stable. By exclusion of other differences, we hence assume a stabilizing effect of the C-terminal aromatic tyrosine residue. The role of tyrosine residues in stabilizing Cu(i) complexes has been previously documented, particularly in amyloidogenic fragments of the chicken prion protein.^67,68^

### 3.4 Secondary structural elements in full-length Ylip_MT and Y54C_Ylip_MT

**Fig. 7:**
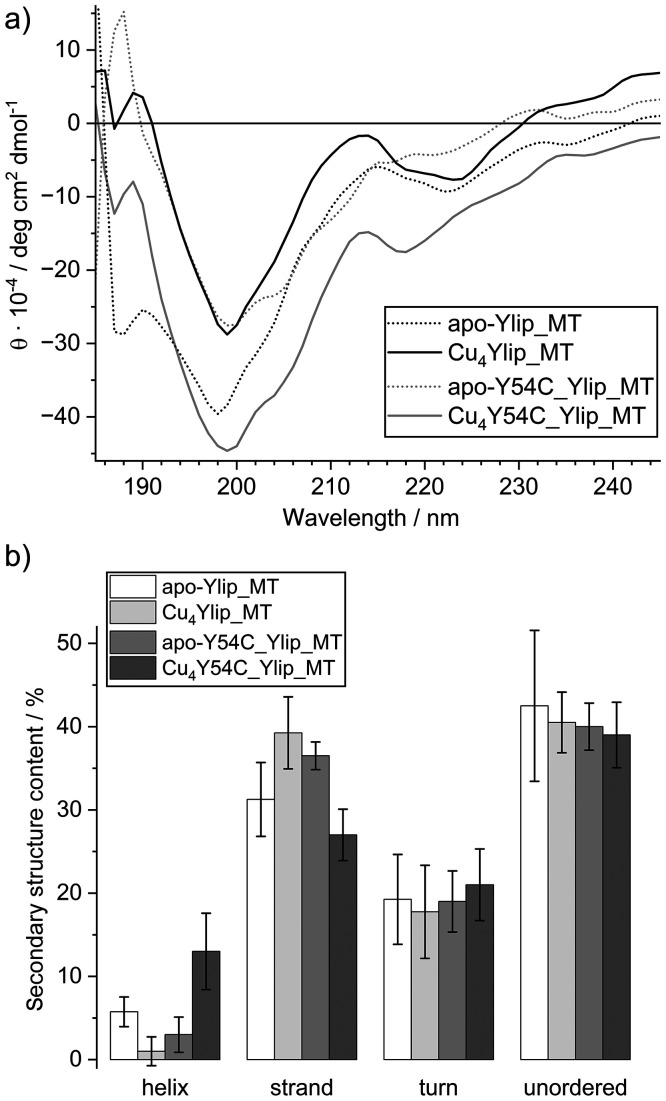
(a) Far-UV CD spectra of full-length Ylip_MT and Y54C_Ylip_MT and their Cu(i) complexes. A protein concentration of 50 µM in 10 mM Tris-HCl 7.4 and 10 mM NaCl. (b) Average secondary structure contents of full-length Ylip_MT and Y54C_Ylip_MT and their Cu(i) complexes, as determined by analysis of the far-UV CD spectra using the CDSSTR algorithm on DichroWeb (reference data sets 3, 4, 5, and SP175t; wavelength range 185–260 nm, Tables S8–S11).

MTs generally contain only limited regions capable of forming well-defined secondary structure elements such as α-helices or β-sheets. Consequently, their overall folding is strongly dependent on metal ion coordination. While numerous three-dimensional structures of mammalian MTs have been reported, structural information on MTs from other organisms, including fungal MTs, remains scarce. Ylip_MT is of particular interest in this context because it contains an unusually long cysteine-free N-terminal segment. At approximately 30 amino acids, this region accounts for more than half of the protein length and raises the question of whether it contributes stable secondary structure elements independent of metal binding. To address this, we analyzed the secondary structure of full-length apo-Ylip_MT and apo-Y54C-Ylip_MT as well as their corresponding Cu(i)-loaded complexes using far-UV circular dichroism (CD) spectroscopy (Fig. 7). The CD spectra were subsequently deconvoluted using the DichroWeb server, employing the CDSSTR algorithm and reference data sets 3, 4, 5, and SP175t (wavelength range 185–260 nm, step size 1 nm; Fig. 7a, Tables S8–S11).^69–71^

Interestingly, the CD spectra of the apo-forms closely resemble those of the Cu(i)-bound species. All samples display a pronounced negative ellipticity band at 198–199 nm, accompanied by weaker negative contributions in the 218–224 nm range. These spectral features indicate a substantial degree of structural disorder that is largely retained upon metal binding.

Deconvolution of the spectra allows assignment of the observed signals to different secondary structure elements. A strong negative band near 200 nm is characteristic of random coils, whereas α-helices exhibit negative bands at 208 and 220 nm with a positive band near 190 nm. β-sheets are associated with a negative band around 218 nm and a positive band near 195 nm, while β-turns show a strong negative band near 191 nm and a positive band around 207 nm.^72,73^ The calculated secondary structure contents indicate that all four samples contain approximately 40% unstructured regions and around 20% β-turns. The α-helical content is generally low (<6%), with the notable exception of Cu_4_Y54C_Ylip_MT, which shows an increased α-helical contribution of ∼13%. The β-strand content varies between 27% and 39%, with the lowest value observed for Cu_4_Y54C_Ylip_MT (Fig. 7b).

Our F-AAS, ESI-MS, and UV spectroscopic analyses demonstrate that both full-length proteins and their truncated variants bind the same number of Cu(i) ions, indicating that metal coordination occurs exclusively through the cysteine residues located in the C-terminal region. In analogy to previously characterized MT structures, this cysteine-rich C-terminal domain is expected to remain largely flexible to accommodate Cu(i)-thiolate cluster formation with minimal steric strain. Such a structural requirement is consistent with the observed enrichment in random coil and β-turn conformations. Consequently, the observed content of well-ordered secondary structure elements detected by CD is most plausibly attributed to the cysteine-free N-terminal region.

Within this framework, the strong similarity between the apo and Cu(i)-bound spectra becomes readily understandable: Cu(i) binding primarily reorganizes the flexible C-terminal cluster-forming region, while leaving the secondary structural elements of the N-terminal region largely unaffected. Thus, the CD data support a modular organization of Ylip_MT, consisting of a structurally flexible, metal-binding C-terminal domain and a partially structured N-terminal region that is largely independent of metal coordination.

### 3.5 Dynamic light scattering and [^15^N,^1^H]-HSQC spectra

To further address the potential structural influence of Cu(i) coordination to Ylip_MT, the hydrodynamic radii (*r*_H_) of full-length apo-Ylip_MT and Cu_4_Ylip_MT were measured using dynamic light scattering (DLS). The results, 2.07 ± 0.07 nm and 2.09 ± 0.08 nm, respectively, are identical within the error limits. These findings are consistent with the far-UV CD analyses, which revealed very similar secondary structure contents irrespective of metal binding. The data further indicates that Cu(i) binding to the cysteine thiolates does not significantly alter the overall size of the protein.

To complement these results with 3D structural data, ^15^N-labeled samples of Ylip_MT, Y54C-Ylip_MT, C-term-Ylip_MT, and C-term-Y54C-Ylip_MT were prepared and [^15^N,^1^H]-HSQC spectra recorded (data not shown). However, the observed signal dispersion and line widths suggest the presence of conformational exchange processes. Only the spectrum of Cu_4_-Y54C-Ylip_MT shows a modest degree of dispersion, but even in this case, the data were insufficient for further structure calculations (Fig. S11).

## 4. Conclusions

In this study, we set out to elucidate how specific sequence features influence Cu(i) coordination in fungal metallothioneins, with a particular focus on the role of the cysteine-free N-terminal domain, the unique CCC motif, and the absence of a conserved C-terminal cysteine residue in *Yarrowia lipolytica* MT. To address this, we performed a detailed investigation of Cu(i) binding to full-length Ylip_MT, the Y54C variant, and their respective C-terminal domains using complementary optical spectroscopic and potentiometric approaches.

While numerous studies have determined stepwise Cu(i) binding constants for human metallothioneins, employing competition assays with chromophoric ligands,^74^ ESI-MS with competing ligands,^75^ isothermal titration calorimetry,^76,77^ and simulations based on high-resolution MS data,^29^ direct potentiometric analysis of Cu(i)-MT systems was missing. Only recently has potentiometry been applied to a small cysteine-rich Cu(i)-binding motif from a CopY repressor.^60^ To the best of our knowledge, the present work represents the first successful application of potentiometric titrations to a Cu(i)-MT system, enabling direct access to protonation equilibria and stepwise metal-binding constants in these highly cysteine-rich proteins.

By combining spectroscopic evidence with thermodynamic data, we arrive at the following key conclusions:

(i) Maximum Cu(i) loading and cluster formation. All four constructs bind up to four Cu(i) ions. Both UV-vis spectroscopy and potentiometric data consistently support the formation of a Cu_4_Cys_8–9_ cluster. Importantly, this implies that all cysteine residues, including those within the CCC motif, participate in coordination. Thus, the initially raised question regarding the involvement of the complete CCC stretch can be answered affirmatively.

(ii) Functional role of the N-terminal segment. Comparison of full-length proteins with their isolated C-terminal domains demonstrates that the long, cysteine-free N-terminal region enhances Cu(i) binding and stabilizes the resulting complexes. Across the investigated pH range, the Cu_4_ complex of full-length Ylip_MT shows the highest thermodynamic stability. While it does not directly contribute thiolate ligands, the N-terminal segment clearly modulates the stability of the metal-binding core. Significantly decreased p*K*_a_ values in full-length Ylip_MT and its C-terminal fragment strongly support coordination of Cu(i) by the N-terminal amino group.

(iii) Influence of the C-terminal tyrosine residue. In the truncated constructs, the C-terminal tyrosine in the wild-type fragment appears to contribute to complex stabilization at near-physiological pH. While the Y54C variant exhibits slightly higher Cu(i) binding efficiency under strongly acidic conditions (up to pH 3.5), the Cu_4_ complex of the wild-type C-terminal fragment becomes thermodynamically more stable at higher pH. This suggests that the aromatic residue may provide additional stabilization, possibly through secondary interactions that become relevant once the thiolate cluster is largely formed.

Overall, this work establishes potentiometry as a powerful tool for dissecting Cu(i) coordination in MTs and provides new insights into how subtle sequence features such as CCC motifs, terminal residues, and non-coordinating extensions fine-tune Cu(i) cluster formation and stability.

## Conflicts of interest

There are no conflicts to declare.

## Supplementary Material

DT-055-D5DT00689A-s001

## Data Availability

The authors declare that all data underlying the results are available as part of the main article and the data supporting this article have been included as part of the supplementary information (SI). Supplementary information is available. See DOI: https://doi.org/10.1039/d5dt00689a.

## References

[cit1] Blindauer C. A., Leszczyszyn O. I. (2010). Nat. Prod. Rep..

[cit2] Schicht O., Freisinger E. (2009). Inorg. Chim. Acta.

[cit3] Roesijadi G. (1996). Comp. Biochem. Physiol., Part C: Pharmacol., Toxicol. Endocrinol..

[cit4] Vašák M., Hasler D. W. (2000). Curr. Opin. Chem. Biol..

[cit5] Freisinger E., Vašák M. (2013). Met. Ions Life Sci..

[cit6] Feng W., Benz F. W., Cai J., Pierce W. M., Kang Y. J. (2006). J. Biol. Chem..

[cit7] Maret W. (2000). J. Nutr..

[cit8] Reinecke F., Levanets O., Olivier Y., Louw R., Semete B., Grobler A., Hidalgo J., Smeitink J., Olckers A., Van der Westhuizen F. H. (2006). Biochem. J..

[cit9] Hassinen V. H., Tervahauta A. I., Schat H., Karenlampi S. O. (2011). Plant Biol..

[cit10] Braun W., Vašák M., Robbins A. H., Stout C. D., Wagner G., Kägi J. H. R., Wüthrich K. (1992). Proc. Natl. Acad. Sci. U. S. A..

[cit11] Kojima Y. , Binz P.-A. and Kägi J. H. R., in Metallothionein IV, ed. C. Klaassen, Birkhäuser Verlag, Basel, 1st edn, 1999, pp. 3–6

[cit12] Kojima Y. , in Metallobiochemistry Part B Metallothionein and Related Molecules, ed. J. F. Riordan and B. L. Vallee, 1991, vol. 205, pp. 8–10

[cit13] Festa R. A., Thiele D. J. (2011). Curr. Biol..

[cit14] Binesh A., Venkatachalam K. (2024). J. Biochem. Mol. Toxicol..

[cit15] Banu B. S., Ishaq M., Danadevi- K., Padmavathi P., Ahuja Y. R. (2004). Food Chem. Toxicol..

[cit16] Rubino J. T., Franz K. J. (2012). J. Inorg. Biochem..

[cit17] Kozlowski H., Janicka-Klos A., Brasun J., Gaggelli E., Valensin D., Valensin G. (2009). Coord. Chem. Rev..

[cit18] Tümer Z., Moller L. B. (2010). Eur. J. Hum. Genet..

[cit19] Chen L. Y., Min J. X., Wang F. D. (2022). Signal Transduction Targeted Ther..

[cit20] Calvo J., Jung H. M., Meloni G. (2017). IUBMB Life.

[cit21] Öhrvik H., Aaseth J., Horn N. (2017). Metallomics.

[cit22] Butt T. R., Sternberg E., Herd J., Crooke S. T. (1984). Gene.

[cit23] Winge D. R., Nielson K. B., Gray W. R., Hamer D. H. (1985). J. Biol. Chem..

[cit24] Luchinat C., Dolderer B., Del Bianco C., Echner H., Hartmann H. J., Voelter W., Weser U. (2003). J. Biol. Inorg. Chem..

[cit25] Calderone V., Dolderer B., Hartmann H. J., Echner H., Luchinat C., Del Bianco C., Mangani S., Weser U. (2005). Proc. Natl. Acad. Sci. U. S. A..

[cit26] Münger K., Lerch K. (1985). Biochemistry.

[cit27] Lerch K. (1980). Nature.

[cit28] Faller P., Vašák M. (1997). Biochemistry.

[cit29] Melenbacher A., Stillman M. J. (2024). Metallomics.

[cit30] Bogumil R., Faller P., Pountney D. L., Vašák M. (1996). Eur. J. Biochem..

[cit31] Meloni G., Faller P., Vašák M. (2007). J. Biol. Chem..

[cit32] Peris-Díaz M. D., Wu S., Mosna K., Liggett E., Barkhanskiy A., Orzel A., Barran P., Krezel A. (2023). Anal. Chem..

[cit33] Consortium T. U. (2025). Nucleic Acids Res..

[cit34] Karp P. D., Billington R., Caspi R., Fulcher C. A., Latendresse M., Kothari A., Keseler I. M., Krummenacker M., Midford P. E., Ong Q., Ong W. K., Paley S. M., Subhraveti P. (2019). Briefings Bioinf..

[cit35] Garcia S., Prado M., Dégano R., Dominguez A. (2002). J. Biol. Chem..

[cit36] Kapust R. B., Tözser J., Copeland T. D., Waugh D. S. (2002). Biochem. Biophys. Res. Commun..

[cit37] Tropea J. E., Cherry S., Waugh D. S. (2009). Methods Mol. Biol..

[cit38] Habjanič J., Zerbe O., Freisinger E. (2018). Metallomics.

[cit39] Pedersen A. O., Jacobsen J. (1980). Eur. J. Biochem..

[cit40] Hendrixson W. S. (1920). J. Am. Chem. Soc..

[cit41] Buck R. P., Rondinini S., Covington A. K., Baucke F. G. K., Brett C. M. A., Camoes M. F., Milton M. J. T., Mussini T., Naumann R., Pratt K. W., Spitzer P., Wilson G. S. (2002). Pure Appl. Chem..

[cit42] Gans P., O'Sullivan B. (2000). Talanta.

[cit43] Gans P., Sabatini A., Vacca A. (1996). Talanta.

[cit44] Alderighi L., Gans P., Ienco A., Peters D., Sabatini A., Vacca A. (1999). Coord. Chem. Rev..

[cit45] Perinelli M., Tegoni M., Freisinger E. (2020). Inorg. Chem..

[cit46] Pountney D. L., Schauwecker I., Zarn J., Vašák M. (1994). Biochemistry.

[cit47] Roschitzki B., Vašák M. (2002). J. Biol. Inorg. Chem..

[cit48] Tarasava K., Loebus J., Freisinger E. (2016). Int. J. Mol. Sci..

[cit49] Wan X., Schicht O., Freisinger E. (2013). Z. Anorg. Allg. Chem..

[cit50] Hasler D. W., Faller P., Vašák M. (1998). Biochemistry.

[cit51] Bofill R., Capdevila M., Cols N., Atrian S., Gonzàlez-Duarte P. (2001). J. Biol. Inorg. Chem..

[cit52] Rupp H., Weser U. (1978). Biochim. Biophys. Acta.

[cit53] Grimsley G. R., Scholtz J. M., Pace C. N. (2009). Protein Sci..

[cit54] Krzywoszynska K., Swiatek-Kozlowska J., Potocki S., Ostrowska M., Kozlowski H. (2020). J. Inorg. Biochem..

[cit55] Witkowska D., Bielinska S., Kamysz W., Kozlowski H. (2011). J. Inorg. Biochem..

[cit56] Potocki S., Rowinska-Zyrek M., Valensin D., Krzywoszynska K., Witkowska D., Luczkowski M., Kozlowski H. (2011). Inorg. Chem..

[cit57] Rowinska-Zyrek M., Valensin D., Szyrwiel L., Grzonka Z., Kozlowski H. (2009). Dalton Trans..

[cit58] Roos G., Foloppe N., Messens J. (2013). Antioxid. Redox Signaling.

[cit59] Van Laer K., Oliveira M., Wahni K., Messens J. (2014). Protein Sci..

[cit60] Hecel A., Kola A., Dominguez-Martin A., Valensin D. (2025). Inorg. Chem..

[cit61] Harris T. K., Turner G. J. (2002). IUBMB Life.

[cit62] Watly J., Hecel A., Wieczorek R., Swiatek-Korlowska J., Kozlowski H., Rowinska-Zyrek M. (2019). Dalton Trans..

[cit63] Freisinger E. (2007). Inorg. Chim. Acta.

[cit64] Loebus J., Leitenmaier B., Meissner D., Braha B., Krauss G.-J., Dobritzsch D., Freisinger E. (2013). J. Inorg. Biochem..

[cit65] Krezel A., Maret W. (2021). Chem. Rev..

[cit66] Konermann L. , in Encyclopedia of Life Sciences, John Wiley & Sons, Ltd, Chichester, UK, 2012, pp. 1–7. 10.1002/9780470015902.a0003004.pub2

[cit67] Hecel A., Draghi S., Valensin D., Kozlowski H. (2017). Dalton Trans..

[cit68] Hecel A., Valensin D., Kozlowski H. (2019). J. Inorg. Biochem..

[cit69] Compton L. A., Johnson W. C. (1986). Anal. Biochem..

[cit70] Manavalan P., Johnson W. C. (1987). Anal. Biochem..

[cit71] Sreerama N., Woody R. W. (2000). Anal. Biochem..

[cit72] Greenfield N., Fasman G. D. (1969). Biochemistry.

[cit73] Brahms S., Brahms J., Spach G., Brack A. (1977). Proc. Natl. Acad. Sci. U. S. A..

[cit74] Calvo J. S., Lopez V. M., Meloni G. (2018). Metallomics.

[cit75] Banci L., Bertini I., Ciofi-Baffoni S., Kozyreva T., Zovo K., Palumaa P. (2010). Nature.

[cit76] Mehlenbacher M. R., Elsiesy R., Lakha R., Villones R. L. E., Orman M., Vizcarra C. L., Meloni G., Wilcox D. E., Austin R. N. (2022). Chem. Sci..

[cit77] Quinn C. F., Wilcox D. E. (2024). Metallomics.

