# Supplementary material for: Uncovering the copper(i) binding abilities of a unique fungal metallothionein: characterization of Yarrowia lipolytica MT and its Y54C mutant
Source: Dalton Trans. 2026 Apr 13;55(18):7260–73. doi: 10.1039/d5dt00689a (PMC13107397; doi:10.1039/d5dt00689a)
Supplement: DT-055-D5DT00689A-s001 [file DT-055-D5DT00689A-s001.pdf]

Supplementary Information (SI) for Dalton Transactions.
This journal is © The Royal Society of Chemistry 2026

Supplementary Information

to

Uncovering the copper(I) binding abilities of a unique fungal metallothionein: Characterization of *Yarrowia lipolytica* MT and its Y54C mutant

Aleksandra Hecel,[a,b] Mariano Briccola,[b] Eva Freisinger[b*]

[a] Faculty of Chemistry, University of Wrocław, 50-383 Wrocław, Poland

[b] Department of Chemistry, University of Zurich, Zurich, Switzerland
E-mail: freisinger@chem.uzh.ch

Table of contents

Figure S1	Locations of the five MT genes in *Yarrowia lipolytica*	S 3
Figure S2	Codon optimization of Ylip_MT sequence for expression in *E. coli*	S 4
Figure S3	Primers to construct Y54C_Ylip_MT by point mutation	S 4
Figure S4	MS spectra of the Cu(I) complexes	S 5
Figure S5	UV-titration of C-term_Ylip_MT and C-term_Y54C_Ylip_MT with Cu(I)	S 8
Table S1	Fitting results of ε_{262nm} plots from Figures 2 and S5 using the Hill equation	S 9
Table S2	Number of Cys-S^{-} ligands for Ylip_MT and Y54C_Ylip_MT from the ε_{262nm} plots	S 9
Table S3	Number of Cys-S^{-} ligands for the two truncated constructs from the ε_{262nm} plots	S 9
Figure S6	CD-titration of Ylip_MT and C-term_Ylip_MT with Cu(I)	S 10
Figure S7	CD-titration of Y54C_Ylip_MT and C-term_Y54C_Ylip_MT with Cu(I)	S 11
Figure S8	Potentiometric pH titration curves	S 12
Table S4	Protonation constants of apo-Ylip_MT	S 13
Data S1	Discussion of protonation constant data in Table S4	S 13
Table S5	Protonation constants of C-term_Ylip_MT upon Cu(I) addition	S 14
Table S6	Protonation constants of C-term_Y54C_Ylip_MT upon Cu(I) addition	S 15
Table S7	Protonation constants of Ylip_MT upon Cu(I) addition	S 16
Data S2	Discussion of protonation constant data in Table S7	S 16
Figure S9	Species distribution diagrams of the C-terminal constructs in presence of Cu(I)	S 18
Figure S10	Species distribution diagrams of Ylip_MT in presence of Cu(I)	S 19
Table S8	Analysis of the apo-Ylip_MT CD spectrum with DichroWeb	S 19
Table S9	Analysis of the apo-Y54C_Ylip_MT CD spectrum with DichroWeb	S 20
Table S10	Analysis of the Cu_4Ylip_MT CD spectrum with DichroWeb	S 20
Table S11	Analysis of the Cu_4Y54C_Ylip_MT CD spectrum with DichroWeb	S 20
Figure S11	Overlay of [^{15}N, ^{1}H]-HSQC spectra of apo-Y54C_Ylip_MT and Cu_4Y54C_Ylip_MT	S 21
References		S 21

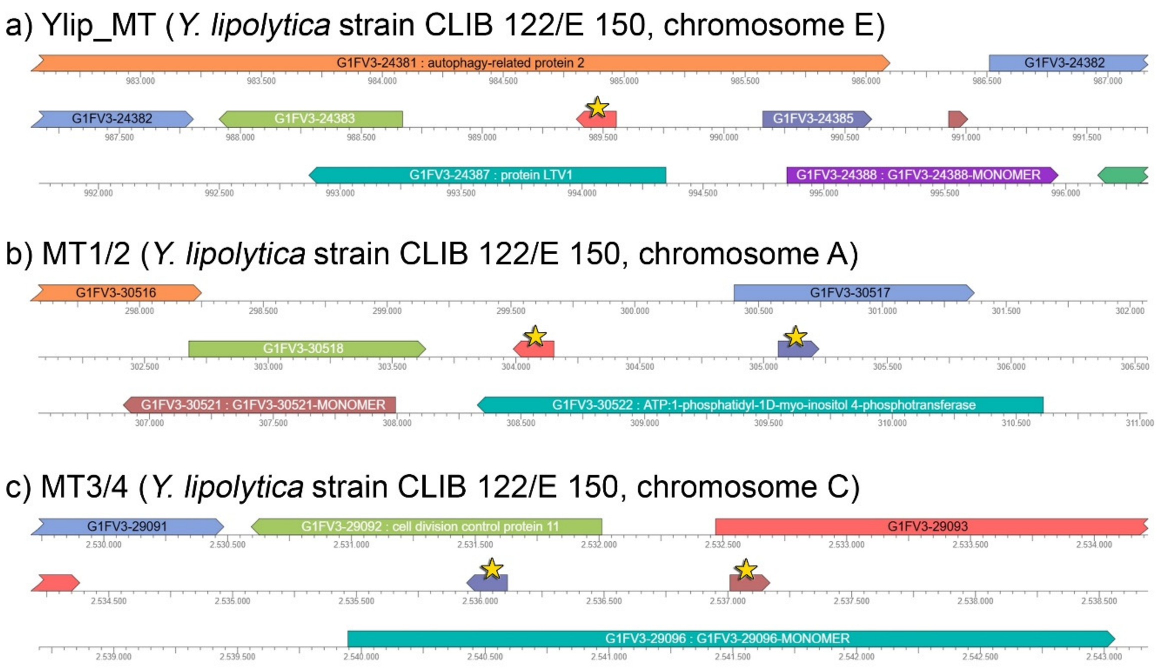

Figure S1. Locations of the five MT genes in *Yarrowia lipolytica* strain CLIB 122/E 150, highlighted by a yellow star, respectively. a) YLIP_MT is located at position 989’391-989’558, b) MT1 at 303’990-304’157, MT2 at 305’061-305’225, c) MT3 at 2’535’946-2’536’113, and MT4 at 2’537’010-2’537’174. The genomic map was produced with The BioCyc Genome Explorer.[1, 2]

	GGA	TTC	TCA	TCT	GCC	ATG	TTT
ACTTGGATCCGAAAACCTGTACTTTCAA	GGA	TTT	TCC	TCG	GCC	ATG	TTT
BamHI TEV cleavage site	G	F	S	S	A	M	F
	GGA	GCT	TCT	CTC	ATC	TCC	AAC
	GGT	GCG	TCC	TTA	ATT	AGC	AAC
	G	A	S	L	I	S	N
	TCC	ATC	TCC	TCT	GAG	TCC	AAC
	TCT	ATC	TCC	TCC	GAA	AGT	AAT
	S	I	S	S	E	S	N
	TTC	AAC	AAA	CGA	GAC	TTG	GTC
	TTT	AAT	AAG	CGT	GAT	TTG	GTG
	F	N	K	R	D	L	V
	AAC	AAC	TGC	TGC	TGC	TCG	TCC
	AAT	AAC	TGT	TGT	TGT	TCC	TCT
	N	N	C	C	C	S	S
	CAA	AAC	ATG	CCC	GAC	ACC	TGT
	CAA	AAT	ATG	CCC	GAT	ACT	TGT
	Q	N	M	P	D	T	C
	GCT	TGC	AAA	AAG	TGC	GGC	TGC
	GCT	TGT	AAA	AAA	TGT	GGA	TGT
	A	C	K	K	C	G	C
	AAG	GCC	TGC	AAG	TAC	TAA	
	AAG	GCG	TGC	AAA	TAC	TAA	CCCGGG
	K	A	C	K	Y	stop	XmaI

Figure S2. Codon optimization of Ylip_MT nucleotide sequence for expression in *E. coli*. Top row: native sequence, middle row: optimized sequence with changes in red, bottom row: amino acid sequence. In Addition, the TEV cleavage site is shown in orange and the restriction sites in blue (XmaI) and green (BamHI).

Y55C_F 5'-GTA AGG CGT GCA AAT GCT AAC CCG GGT CGA C-3'
Y55C_R 5'-GTC GAC CCG GGT TAG CAT TTG CAC GCC TTA C-3'

Figure S3. Primers to construct Y54C_Ylip_MT by point mutation.

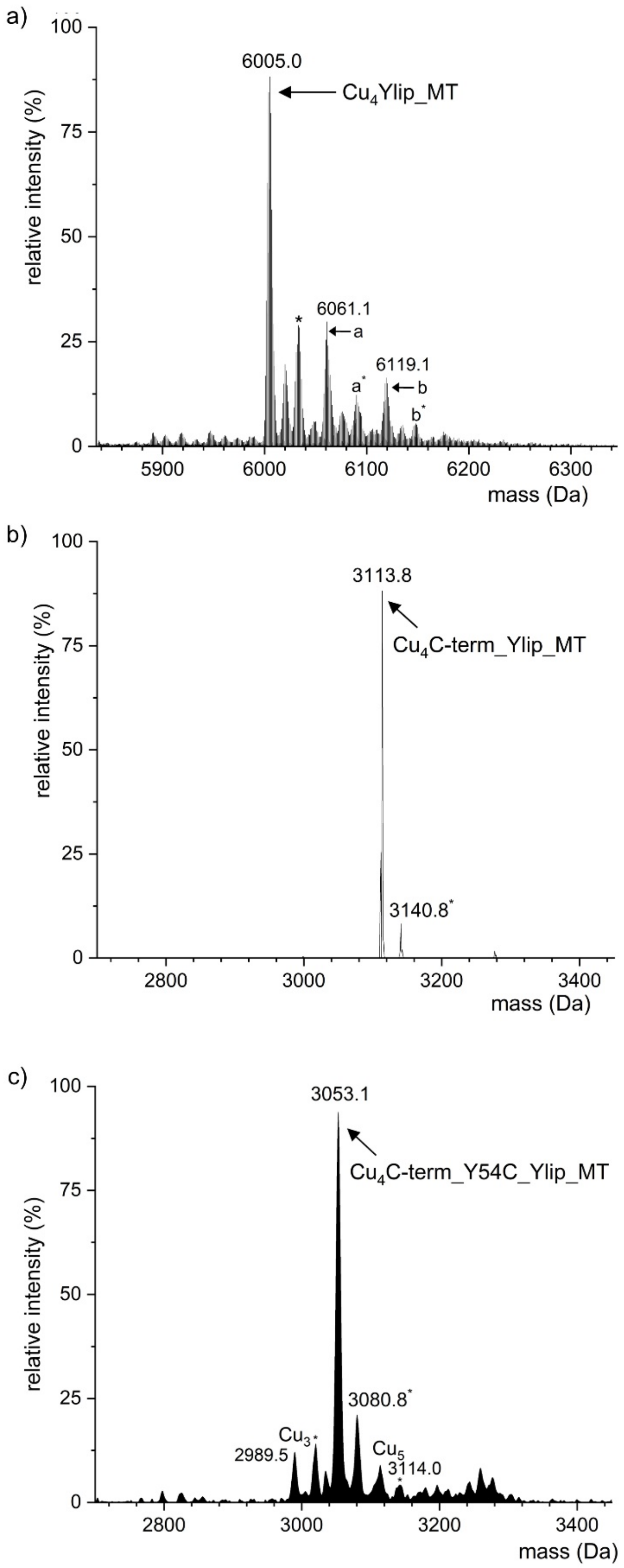

Figure S4.1. a,b) Deconvoluted ESI-MS and c) MALDI-TOF MS spectra of the Cu(I) complexes in the positive ion mode. Mass peaks marked with an asterisk denote +27 Da mass adducts assigned to protein formylation. a) Cu_4Ylip_MT (M_{calc} ($[M+H]^+$) 6005.1 Da), a and b denote unassigned protein adducts from peptide synthesis (see Figure S4.3 for the MS after synthesis); b) Cu_4C-term_Ylip_MT (M_{calc} ($[M+H]^+$) 3113.7 Da); c) Cu_4C-term_Y54C_Ylip_MT (M_{calc} ($[M+H]^+$) 3053.7 Da).

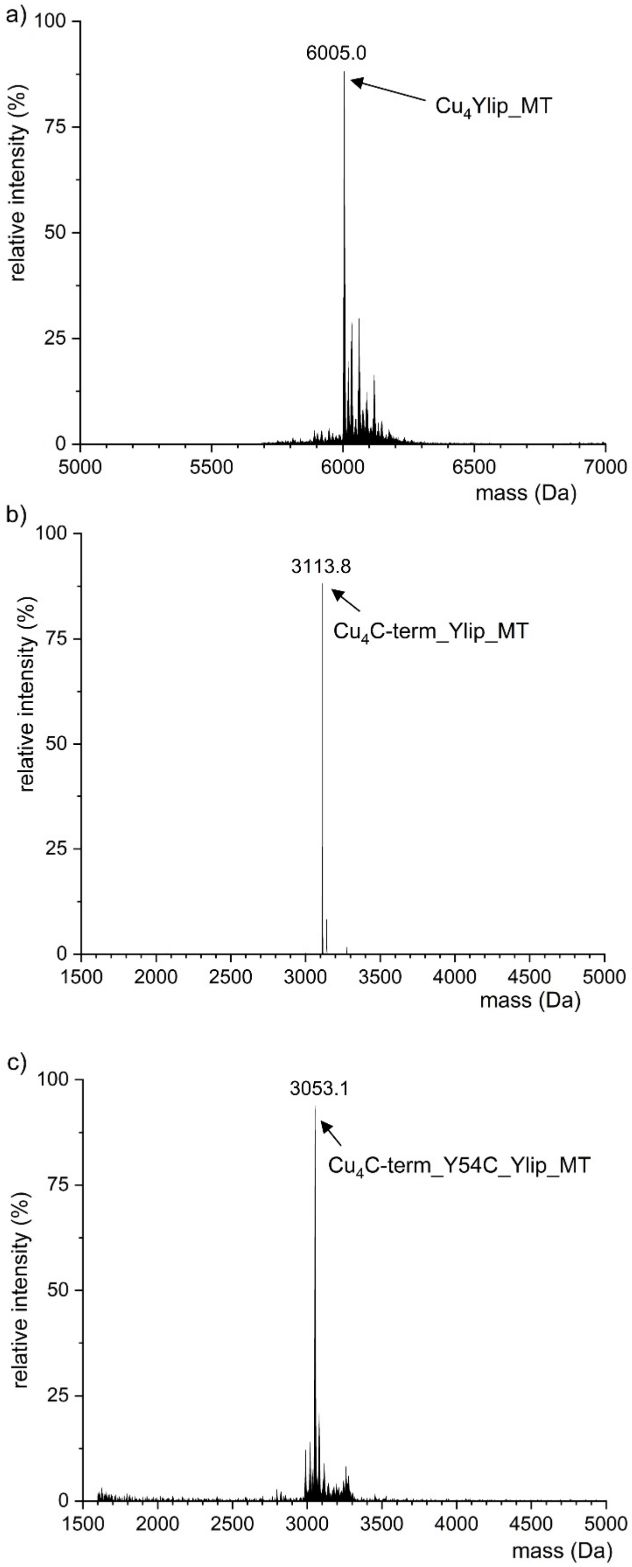

Figure S4.2. Full range deconvoluted ESI-MS (a,b) and MALDI-TOF MS (c) spectra of the Cu(I) complexes in the positive ion mode. a) Cu_4Ylip_MT (M_{calc} ($[M+H]^+$) 6005.1 Da), b) Cu_4C-term_Ylip_MT (M_{calc} ($[M+H]^+$) 3113.7 Da), c) Cu_4C-term_Y54C_Ylip_MT (M_{calc} ($[M+H]^+$) 3053.7 Da).

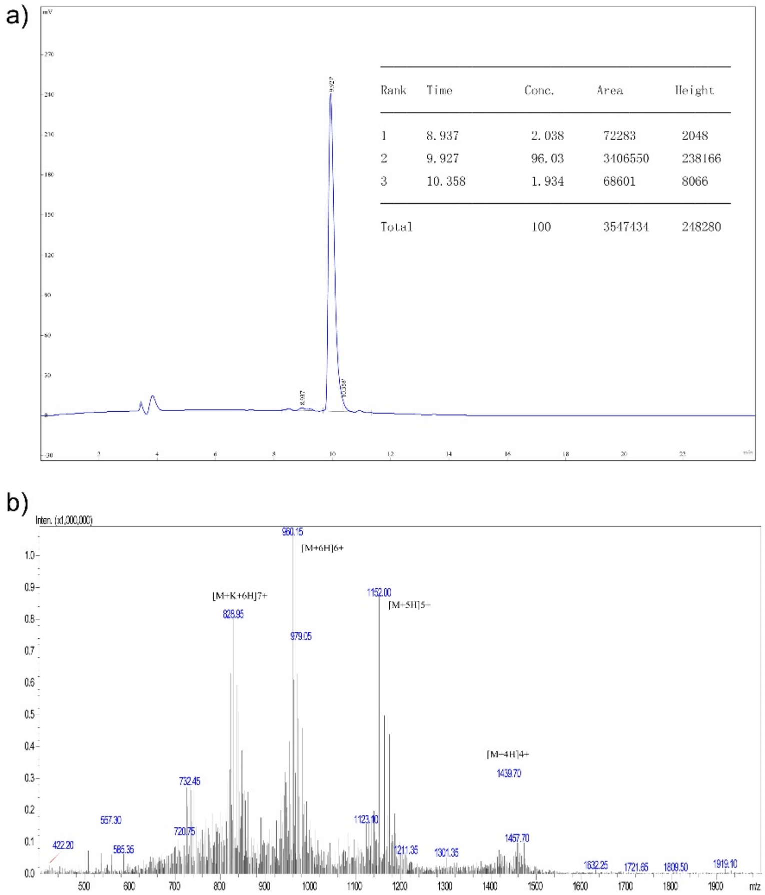

Figure S4.3. Analysis report of full-lengths Ylip_MT as received from GL Biochem Ltd. (Shanghai, P.R. China). a) HPLC trace (0.1 % TFA in acetonitrile/water), b) non-deconvoluted ESI-MS spectrum in the positive ion mode.

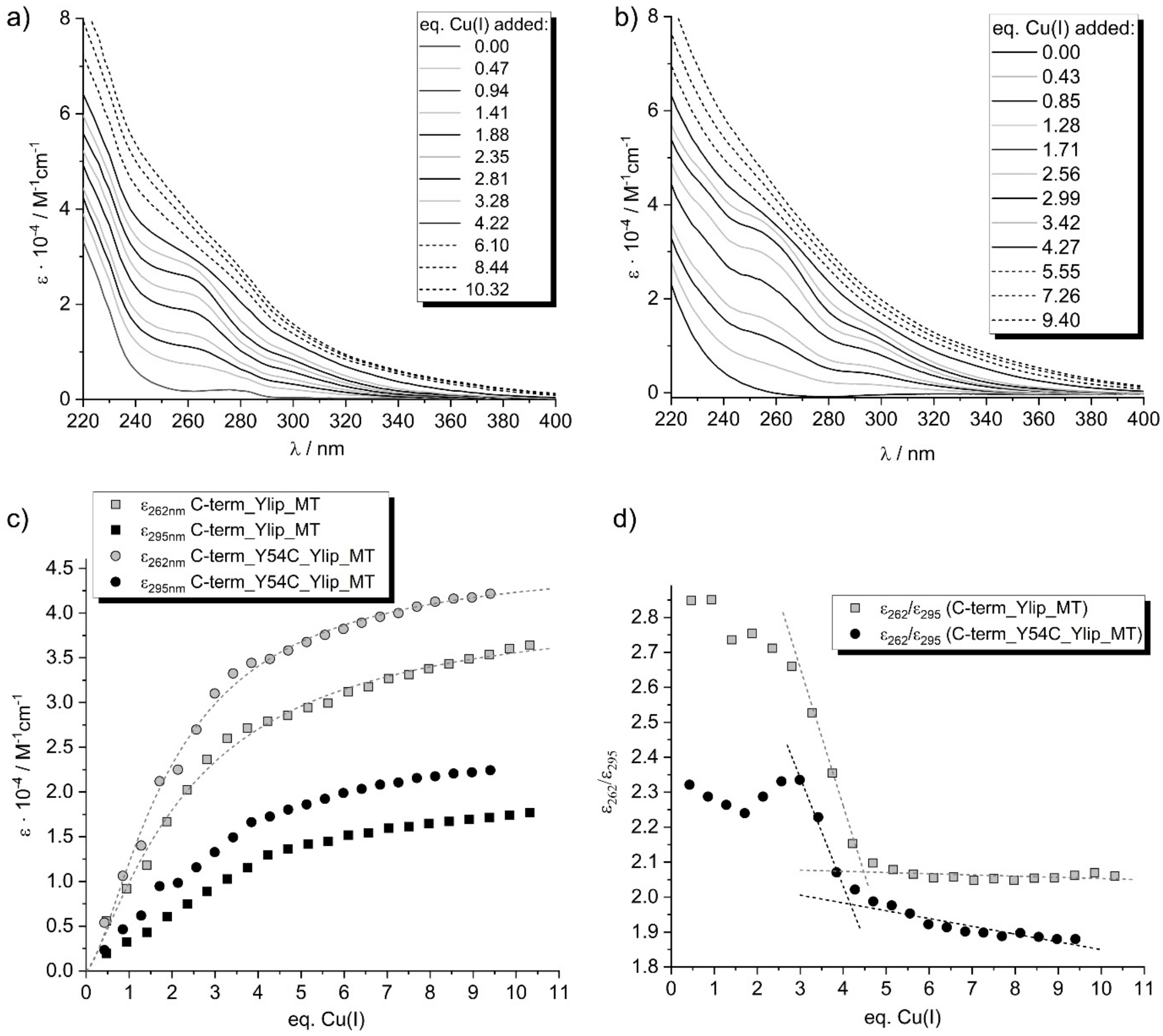

Figure S5. Titration of C-term_Ylip_MT and C-term_Y54C_Ylip_MT with Cu(I) monitored with UV spectroscopy. a) Selected UV spectra for C-term_Ylip_MT and b) selected UV spectra for C-term_Y54C_Ylip_MT. c) Plots of molar absorptivity values at 262 nm and 295 nm for both proteins, including data fitting with the *Hill equation* for the ε_{262} data (dotted lines, see Table S1). d) $\varepsilon_{262}/\varepsilon_{295}$ ratio for both proteins, including linear fits of data to estimate the maximum Cu(I) binding capacity (protein concentration 8-15 μM, in 10 mM Tris-HCl 7.6 and 10 mM NaCl).

Table S1 Fitting results of ε_{262nm} plots from Figures 2 and S5 using the Hill equation:

$$\varepsilon_{262} = \varepsilon_{262,\max} \cdot \frac{\mathrm{x}^n}{\mathrm{K}_\mathrm{A}^n + \mathrm{x}^n}$$

with K_A : [Cu(I)] producing ½ $\varepsilon_{262,max}$ *n* : *Hill coefficient*

	Ylip_MT	Y54C_Ylip_MT	C-term_ Ylip_MT	C-term_ Y54C_Ylip_MT
$\varepsilon_{262,max}$	34828 ± 1022	38975 ± 1105	41324 ± 1343	461456 ± 987
K_A	2.16 ± 0.11	2.01 ± 0.10	2.45 ± 0.15	2.00 ± 0.08
n	1.45 ± 0.08	1.50 ± 0.08	1.29 ± 0.08	1.49 ± 0.08
R^2	0.99612	0.99585	0.99303	0.99462

Table S2 Calculated number of coordinating Cys-S⁻ ligands using the curve fitting parameters from Table S1 and an ε_{262} value per Cu(I)-bound Cys-S⁻ derived from the calculated ε_{262} value at 4.0 or 3.0 eq. Cu(I), respectively, considering eight Cys-S⁻ for Ylip_MT and nine Cys-S⁻ for Y54C_Ylip_MT.

	Ylip_MT			Y54C_Ylip_MT		
eq. Cu(I)	$\varepsilon_{262,calc}$	*N*(Cys-S⁻)[a]		$\varepsilon_{262,calc}$	*N*(Cys-S⁻)[b]	
0.5	3704.1	1.20	1.38	4407.2	1.38	1.58
1.0	8629.3	2.79	3.21	10152.0	3.18	3.65
1.5	12939.6	4.20	4.82	15209.1	4.77	5.46
2.0	16455.8	5.33	6.13	19293.0	6.05	6.93
2.5	19255.9	6.24	7.17	22515.5	7.06	8.09
3.0	21481.7	6.96	8.00	25056.7	7.86	9.00
3.5	23263.2	7.53		27077.3	8.49	
4.0	24704.1	8.00		28702.4	9.00	

[a] $\varepsilon_{262,calc}$: $\frac{24704.1}{8}$ or $\frac{21481.7}{8}$ [b] $\varepsilon_{262,calc}$: $\frac{28702.4}{9}$ or $\frac{25056.7}{9}$

Table S3 Calculated number of coordinating Cys-S⁻ ligands using the curve fitting parameters from Table S1 and an ε_{262} value per Cu(I)-bound Cys-S⁻ derived from the calculated ε_{262} value at 4.0 or 3.0 eq. Cu(I), respectively, considering eight Cys-S⁻ for C-term_Ylip_MT and nine Cys-S⁻ for C-term_Y54C_Ylip_MT.

	C-term_Ylip_MT			C-term_Y54C_Ylip_MT		
eq. Cu(I)	$\varepsilon_{262,calc}$	*N*(Cys-S⁻)[a]		$\varepsilon_{262,calc}$	*N*(Cys-S⁻)[b]	
0.5	4693.7	1.39	1.61	5168.4	1.37	1.56
1.0	9882.0	2.92	3.38	12096.3	3.20	3.65
1.5	14336.3	4.24	4.91	18196.5	4.81	5.48
2.0	17988.8	5.32	6.16	23080.1	6.10	6.96
2.5	20957.8	6.20	7.17	26890.5	7.11	8.10
3.0	23380.1	6.92	8.00	29861.5	7.89	9.00
3.5	25373.1	7.51		32198.7	8.51	
4.0	27029.2	8.00		34060.4	9.00	

$^{a}\ \varepsilon_{262,calc}: \frac{27029.2}{8}$ or $\frac{23380.1}{8}$ $\qquad$ $^{b}\ \varepsilon_{262,calc}: \frac{34060.4}{9}$ or $\frac{29861.5}{9}$

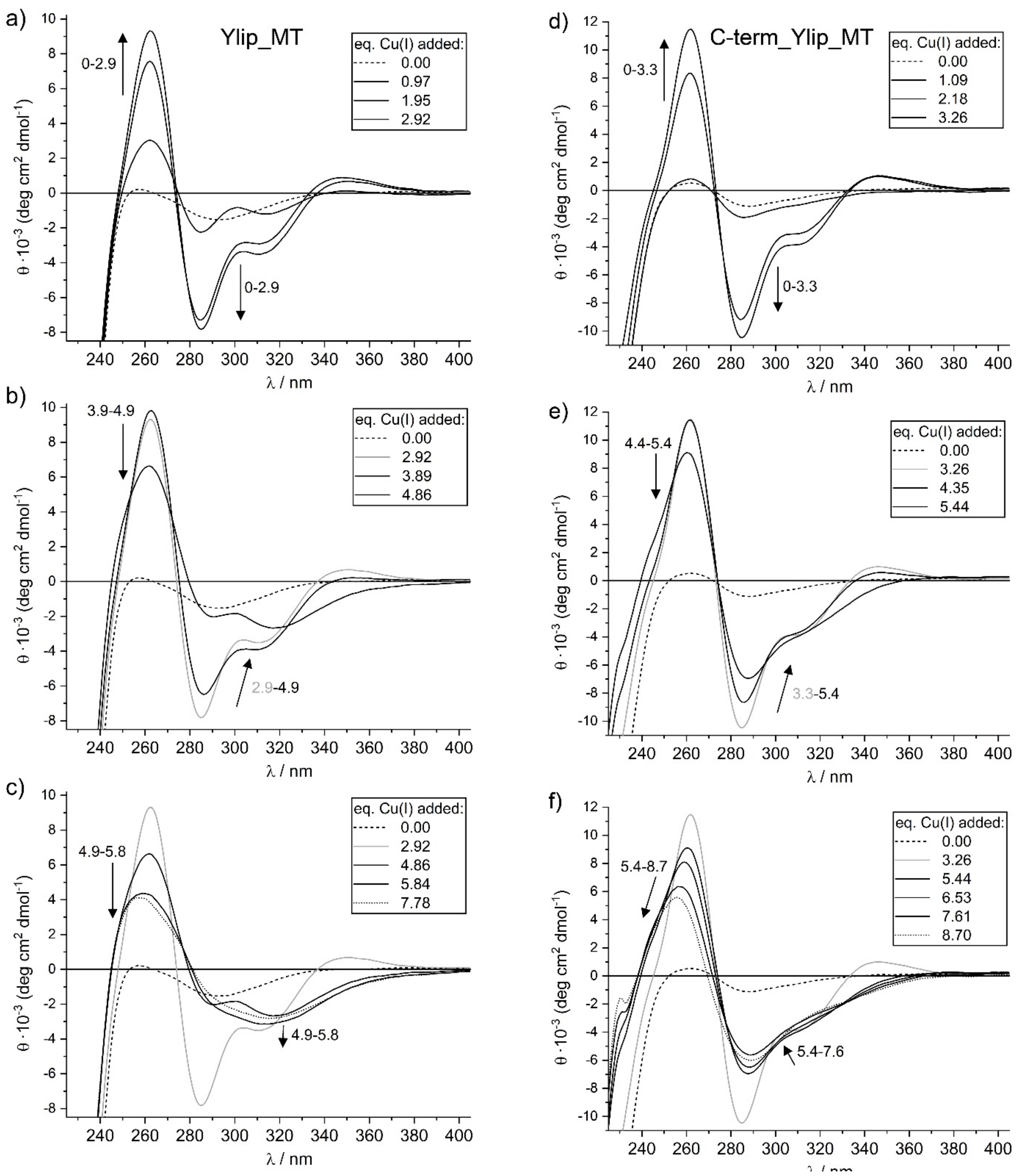

Figure S6. Titration of Ylip_MT (a-c) and C-term_Ylip_MT (d-f) with Cu(I) monitored with CD spectroscopy. Protein concentrations 8-15 μM, in 10 mM Tris-HCl 7.4 and 10 mM NaCl.

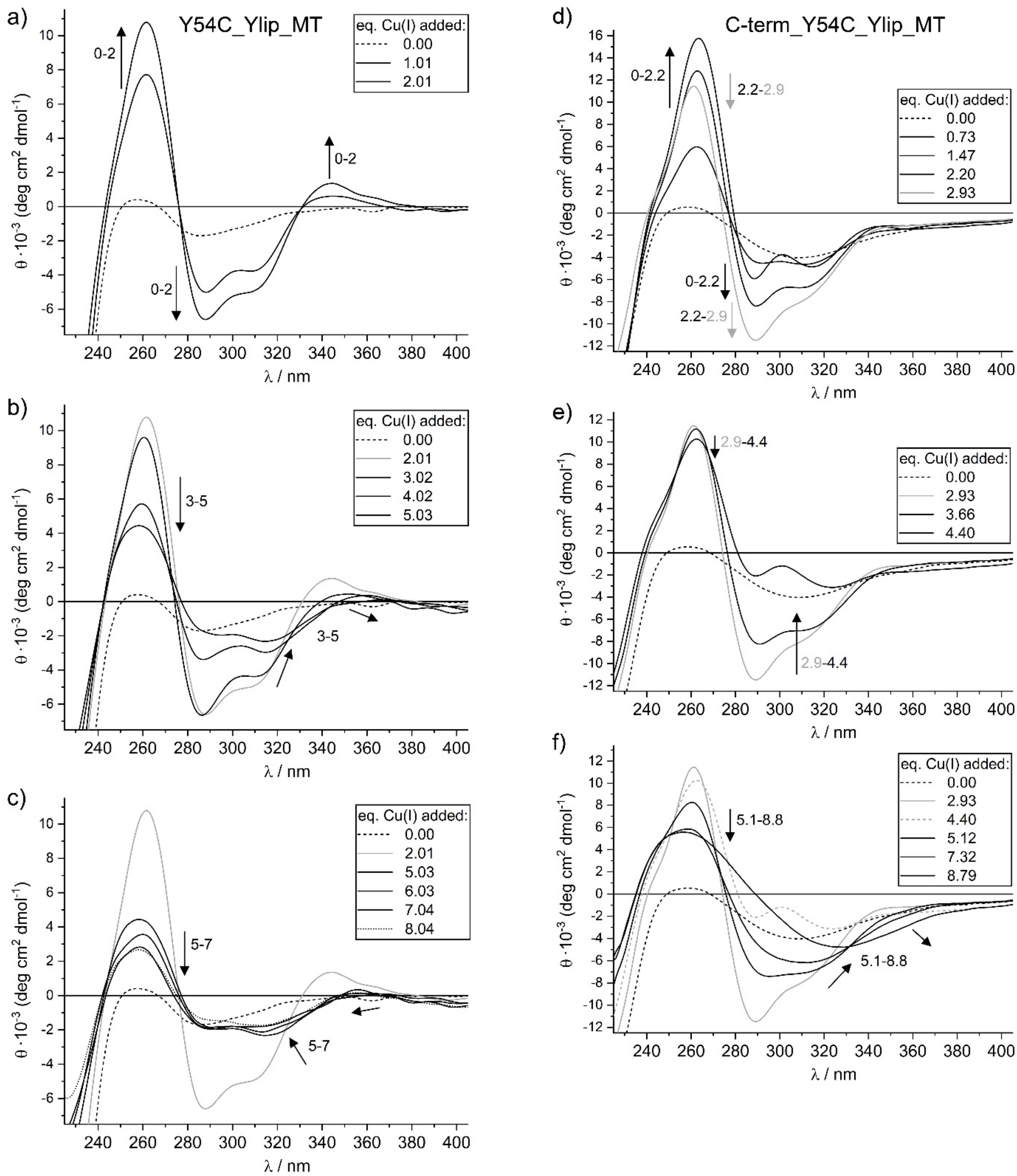

Figure S7. Titration of Y54C_Ylip_MT (a-c) and C-term_Y54C_Ylip_MT (d-f) with Cu(I) monitored with CD spectroscopy. Protein concentrations 8-15 µM, in 10 mM Tris-HCl 7.4 and 10 mM NaCl.

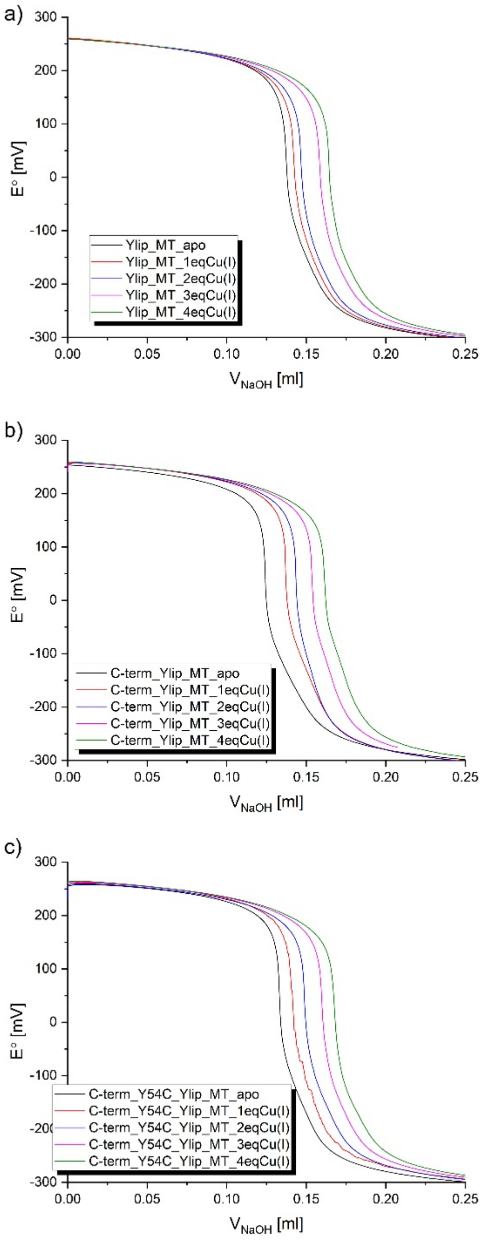

Figure S8. Potentiometric pH titration curves (initial solution: apo-protein in 10 mM HCl in presence of 0-4 equivalents of Cu(I)) at I = 0.1 M KCl, T = 298 K. a) Ylip_MT, b) C-term_Ylip_MT, c) C-term_Y54C_Ylip_MT. See main manuscript for experimental details.

Table S4. Protonation constants of apo-Ylip_MT determined by potentiometric pH titration (initial solution: 10 mM HCl) at I = 0.1 M KCl, T = 298 K. Standard deviations are given in parentheses as uncertainties on the last significant digit.

protonation site	pK_a	log β	species
C-terminus	2.0	83.8(1)	$(H_6L)H_{13}$
Asp-COOH	3.0	81.8(1)	$(H_6L)H_{12}$
Asp-COOH	3.8	78.8(1)	$(H_6L)H_{11}$
Glu-COOH	4.1	75.0(1)	$(H_6L)H_{10}$
Cys-SH	5.1	70.9(1)	$(H_6L)H_9$
Cys-SH	7.2	65.8(1)	$(H_6L)H_8$
Cys-SH	7.4	58.6(1)	$(H_6L)H_7$
Cys-SH	8.0	51.2(1)	$(H_6L)H_6$
Cys-SH	8.0	43.2(1)	$(H_6L)H_5$
Cys-SH	8.6	35.2(1)	$(H_6L)H_4$
Cys-SH	8.7	26.6(1)	$(H_6L)H_3$
Cys-SH	8.9	17.9(1)	$(H_6L)H_2$
N-terminus	9.0	9.0(1)	$(H_6L)H_1$

Data S1. Discussion of calculated pK_a values in Table S4 for apo-Ylip_MT

Ylip_MT contains 19 functional groups that can undergo deprotonation: the carboxylic acid group of the C-terminus, the two aspartic acids and the glutamic acid, the thiol groups of eight cysteines, the amino groups of the N-terminus and the five lysines, as well as the guanidinium group of arginine. In the monitored pH range of 2-9, thirteen protonation constants were established (Table S4) and hence H_6L was defined as most deprotonated species. The remaining six deprotonation constants, probably those for the five lysine residues and the arginine are outside of the investigated pH range, where oxidation processes impeded further calculations. The first four pK_a values between 2.0 and 4.1 correspond to the four carboxylic groups mentioned above. The next eight (pK_a 5.1-9.0) are assigned to the -SH groups of the cysteine residues and the N-terminal amino group. As mentioned in the main text (potentiometry section of C-terminal fragments), it is not possible to distinguished –SH and $–NH_3^+$ groups due to the narrow pH range in which they occur. The first pK_a value (5.1) is again lower than expected for a cysteine residue as observed similarly for the C-terminal fragments. That the value is slightly higher compared to 4.3 and 4.2 for C-term_Ylip_MT and C-term_Y54C-Ylip_MT, respectively, may be due to the larger distance between the N-terminus and the Cys-rich C-terminal region in Ylip_MT compared to the two truncated constructs.

Table S5. Protonation constants of C-term_Ylip_MT in presence of increasing equivalents of Cu(I) determined by potentiometric pH titration (initial solution: 10 mM HCl) at I = 0.1 M KCl, T = 298 K. Standard deviations are given in parentheses as uncertainties on the last significant digit.

<table>
<tr><th>protonation site</th><th>Cu_1-species</th><th>log β</th><th>pK_a</th><th>Cu_2-species</th><th>log β</th><th>pK_a</th><th>Cu_3-species</th><th>log β</th><th>pK_a</th><th>Cu_4-species</th><th>log β</th><th>pK_a</th></tr>
<tr><td>C-terminus</td><td>$(H_4L)H_{11}$</td><td></td><td></td><td>$(H_4L)H_{11}$</td><td></td><td></td><td>$(H_4L)H_{11}$</td><td></td><td></td><td>$(H_4L)H_{11}$</td><td></td><td></td></tr>
<tr><td>Asp-COOH</td><td>$(H_4L)H_{10}$</td><td></td><td></td><td>$(H_4L)H_{10}$</td><td></td><td></td><td>$(H_4L)H_{10}$</td><td></td><td></td><td>$(H_4L)H_{10}$</td><td></td><td></td></tr>
<tr><td rowspan="9">8 x Cys-SH plus N-terminus</td><td>$(H_4L)H_9$</td><td></td><td></td><td>$(H_4L)H_9$</td><td></td><td></td><td>$(H_4L)H_9$</td><td></td><td></td><td>$(H_4L)H_9$</td><td></td><td></td></tr>
<tr><td>$(H_4L)H_8$</td><td></td><td></td><td>$(H_4L)H_8$</td><td></td><td></td><td>$(H_4L)H_8$</td><td></td><td></td><td>$(H_4L)H_8$</td><td></td><td></td></tr>
<tr><td>$(H_4L)H_7$</td><td>67.1(1)</td><td>3.1</td><td>$(H_4L)H_7$</td><td>-</td><td></td><td>$(H_4L)H_7$</td><td>-</td><td></td><td>$(H_4L)H_7$</td><td>-</td><td></td></tr>
<tr><td>$(H_4L)H_6$</td><td>64.0(1)</td><td rowspan="2">3.8</td><td>$(H_4L)H_6$</td><td>67.2(1)</td><td rowspan="2">3.5</td><td>$(H_4L)H_6$</td><td>70.9(1)</td><td rowspan="2">3.2</td><td>$(H_4L)H_6$</td><td>-</td><td></td></tr>
<tr><td>$(H_4L)H_5$</td><td>-</td><td>$(H_4L)H_5$</td><td>-</td><td>$(H_4L)H_5$</td><td>-</td><td>$(H_4L)H_5$</td><td>71.3(1)</td><td rowspan="2">2.95</td></tr>
<tr><td>$(H_4L)H_4$</td><td>56.4(1)</td><td>5.0</td><td>$(H_4L)H_4$</td><td>60.2(1)</td><td rowspan="2">4.2</td><td>$(H_4L)H_4$</td><td>64.5(1)</td><td>3.2</td><td>$(H_4L)H_4$</td><td>-</td></tr>
<tr><td>$(H_4L)H_3$</td><td>51.4(1)</td><td>7.2</td><td>$(H_4L)H_3$</td><td>-</td><td>$(H_4L)H_3$</td><td>61.3(1)</td><td rowspan="2">3.65</td><td>$(H_4L)H_3$</td><td>65.4(1)</td><td rowspan="3">3.23</td></tr>
<tr><td>$(H_4L)H_2$</td><td>44.2(1)</td><td>7.3</td><td>$(H_4L)H_2$</td><td>51.9(1)</td><td>6.2</td><td>$(H_4L)H_2$</td><td>-</td><td>$(H_4L)H_2$</td><td>-</td></tr>
<tr><td>$(H_4L)H_1$</td><td>36.9(1)</td><td>7.5</td><td>$(H_4L)H_1$</td><td>45.7(1)</td><td>7.2</td><td>$(H_4L)H_1$</td><td>54.0(1)</td><td>3.7</td><td>$(H_4L)H_1$</td><td>-</td></tr>
<tr><td></td><td>(H_4L)</td><td>29.4(1)</td><td></td><td>(H_4L)</td><td>38.5(1)</td><td></td><td>(H_4L)</td><td>50.3(1)</td><td></td><td>(H_4L)</td><td>55.7(1)</td><td></td></tr>
</table>

Table S6. Protonation constants of C-term_Y54C_Ylip_MT in presence of increasing equivalents of Cu(I) determined by potentiometric pH titration (initial solution: 10 mM HCl) at I = 0.1 M KCl, T = 298 K. Standard deviations are given in parentheses as uncertainties on the last significant digit.

<table>
<tr><th>protonation site</th><th>Cu_1-species</th><th>log β</th><th>pK_a</th><th>Cu_2-species</th><th>log β</th><th>pK_a</th><th>Cu_3-species</th><th>log β</th><th>pK_a</th><th>Cu_4-species</th><th>log β</th><th>pK_a</th></tr>
<tr><td>C-terminus</td><td>$(H_5L)H_{11}$</td><td></td><td></td><td>$(H_5L)H_{11}$</td><td></td><td></td><td>$(H_5L)H_{11}$</td><td></td><td></td><td>$(H_5L)H_{11}$</td><td></td><td></td></tr>
<tr><td>Asp-COOH</td><td>$(H_5L)H_{10}$</td><td></td><td></td><td>$(H_5L)H_{10}$</td><td></td><td></td><td>$(H_5L)H_{10}$</td><td></td><td></td><td>$(H_5L)H_{10}$</td><td></td><td></td></tr>
<tr><td rowspan="9">8 x Cys-SH
plus
N-terminus

or

9 x Cys-SH</td><td>$(H_5L)H_9$</td><td></td><td></td><td>$(H_5L)H_9$</td><td></td><td></td><td>$(H_5L)H_9$</td><td></td><td></td><td>$(H_5L)H_9$</td><td></td><td></td></tr>
<tr><td>$(H_5L)H_8$</td><td></td><td></td><td>$(H_5L)H_8$</td><td></td><td></td><td>$(H_5L)H_8$</td><td></td><td></td><td>$(H_5L)H_8$</td><td></td><td></td></tr>
<tr><td>$(H_5L)H_7$</td><td>69.8(1)</td><td>3.2</td><td>$(H_5L)H_7$</td><td>-</td><td></td><td>$(H_5L)H_7$</td><td>-</td><td></td><td>$(H_5L)H_7$</td><td>-</td><td></td></tr>
<tr><td>$(H_5L)H_6$</td><td>66.6(1)</td><td>3.7</td><td>$(H_5L)H_6$</td><td>68.8(1)</td><td>3.3</td><td>$(H_5L)H_6$</td><td>73.2(1)</td><td rowspan="2">2.7</td><td>$(H_5L)H_6$</td><td>75.9(1)</td><td rowspan="2">2.6</td></tr>
<tr><td>$(H_5L)H_5$</td><td>62.9(1)</td><td>4.1</td><td>$(H_5L)H_5$</td><td>65.5(1)</td><td>3.4</td><td>$(H_5L)H_5$</td><td>-</td><td>$(H_5L)H_5$</td><td>-</td></tr>
<tr><td>$(H_5L)H_4$</td><td>58.8(1)</td><td>5.4</td><td>$(H_5L)H_4$</td><td>62.1(1)</td><td>4.1</td><td>$(H_5L)H_4$</td><td>67.8(1)</td><td rowspan="2">3.3</td><td>$(H_5L)H_4$</td><td>70.7(1)</td><td>2.9</td></tr>
<tr><td>$(H_5L)H_3$</td><td>53.4(1)</td><td>6.8</td><td>$(H_5L)H_3$</td><td>58.0(1)</td><td>4.4</td><td>$(H_5L)H_3$</td><td>-</td><td>$(H_5L)H_3$</td><td>67.8(1)</td><td rowspan="3">3.3</td></tr>
<tr><td>$(H_5L)H_2$</td><td>46.6(1)</td><td rowspan="2">7.55</td><td>$(H_5L)H_2$</td><td>53.6(1)</td><td>6.3</td><td>$(H_5L)H_2$</td><td>61.2(1)</td><td rowspan="2">3.6</td><td>$(H_5L)H_2$</td><td>-</td></tr>
<tr><td>$(H_5L)H_1$</td><td>-</td><td>$(H_5L)H_1$</td><td>47.3(1)</td><td>7.4</td><td>$(H_5L)H_1$</td><td>-</td><td>$(H_5L)H_1$</td><td>-</td></tr>
<tr><td></td><td>(H_5L)</td><td>31.5(1)</td><td></td><td>(H_5L)</td><td>39.9(1)</td><td></td><td>(H_5L)</td><td>54.0(1)</td><td></td><td>(H_5L)</td><td>57.9(1)</td><td></td></tr>
</table>

Table S7. Protonation constants of Ylip_MT in presence of increasing equivalents of Cu(I) determined by potentiometric pH titration (initial solution: 10 mM HCl) at I = 0.1 M KCl, T = 298 K. Standard deviations are given in parentheses as uncertainties on the last significant digit.

<table>
<tr><th>protonation site</th><th>Cu₁-species</th><th>log β</th><th>pKa</th><th>Cu₂-species</th><th>log β</th><th>pKa</th><th>Cu₃-species</th><th>log β</th><th>pKa</th><th>Cu₄-species</th><th>log β</th><th>pKa</th></tr>
<tr><td>C-terminus</td><td>$(H_6L)H_{13}$</td><td></td><td></td><td>$(H_6L)H_{13}$</td><td></td><td></td><td>$(H_6L)H_{13}$</td><td></td><td></td><td>$(H_6L)H_{13}$</td><td></td><td></td></tr>
<tr><td>Asp-COOH</td><td>$(H_6L)H_{12}$</td><td></td><td></td><td>$(H_6L)H_{12}$</td><td></td><td></td><td>$(H_6L)H_{12}$</td><td></td><td></td><td>$(H_6L)H_{12}$</td><td></td><td></td></tr>
<tr><td>Asp-COOH</td><td>$(H_6L)H_{11}$</td><td></td><td></td><td>$(H_6L)H_{11}$</td><td></td><td></td><td>$(H_6L)H_{11}$</td><td></td><td></td><td>$(H_6L)H_{11}$</td><td></td><td></td></tr>
<tr><td>Glu-COOH</td><td>$(H_6L)H_{10}$</td><td></td><td></td><td>$(H_6L)H_{10}$</td><td></td><td></td><td>$(H_6L)H_{10}$</td><td></td><td></td><td>$(H_6L)H_{10}$</td><td></td><td></td></tr>
<tr><td rowspan="9">8 x Cys-SH plus N-terminus</td><td>$(H_6L)H_9$</td><td>78.8(1)</td><td>3.4</td><td>$(H_6L)H_9$</td><td>81.8(1)</td><td rowspan="2">3.3</td><td>$(H_6L)H_9$</td><td>-</td><td></td><td>$(H_6L)H_9$</td><td>-</td><td></td></tr>
<tr><td>$(H_6L)H_8$</td><td>75.4(1)</td><td>3.4</td><td>$(H_6L)H_8$</td><td>-</td><td>$(H_6L)H_8$</td><td>-</td><td></td><td>$(H_6L)H_8$</td><td>-</td><td></td></tr>
<tr><td>$(H_6L)H_7$</td><td>72.0(1)</td><td>4.1</td><td>$(H_6L)H_7$</td><td>75.2(1)</td><td>3.9</td><td>$(H_6L)H_7$</td><td>79.4(1)</td><td rowspan="2">3.0</td><td>$(H_6L)H_7$</td><td>-</td><td></td></tr>
<tr><td>$(H_6L)H_6$</td><td>67.9(1)</td><td>4.6</td><td>$(H_6L)H_6$</td><td>71.3(1)</td><td>3.9</td><td>$(H_6L)H_6$</td><td>-</td><td>$(H_6L)H_6$</td><td>79.9(1)</td><td rowspan="2">3.0</td></tr>
<tr><td>$(H_6L)H_5$</td><td>63.3(1)</td><td>6.7</td><td>$(H_6L)H_5$</td><td>67.4(1)</td><td>4.2</td><td>$(H_6L)H_5$</td><td>73.4(1)</td><td rowspan="2">3.55</td><td>$(H_6L)H_5$</td><td>-</td></tr>
<tr><td>$(H_6L)H_4$</td><td>56.6(1)</td><td>7.7</td><td>$(H_6L)H_4$</td><td>63.2(1)</td><td>5.0</td><td>$(H_6L)H_4$</td><td>-</td><td>$(H_6L)H_4$</td><td>73.9(1)</td><td rowspan="2">3.4</td></tr>
<tr><td>$(H_6L)H_3$</td><td>48.9(1)</td><td>7.9</td><td>$(H_6L)H_3$</td><td>58.2(1)</td><td>7.1</td><td>$(H_6L)H_3$</td><td>66.3(1)</td><td>4.1</td><td>$(H_6L)H_3$</td><td>-</td></tr>
<tr><td>$(H_6L)H_2$</td><td>41.0(1)</td><td rowspan="2">8.45</td><td>$(H_6L)H_2$</td><td>51.1(1)</td><td>7.5</td><td>$(H_6L)H_2$</td><td>62.2(1)</td><td>4.1</td><td>$(H_6L)H_2$</td><td>67.1(1)</td><td rowspan="2">3.7</td></tr>
<tr><td>$(H_6L)H_1$</td><td>-</td><td>$(H_6L)H_1$</td><td>43.6(1)</td><td>7.9</td><td>$(H_6L)H_1$</td><td>58.1(1)</td><td>4.2</td><td>$(H_6L)H_1$</td><td>-</td></tr>
<tr><td></td><td>(H_6L)</td><td>24.1(1)</td><td></td><td>(H_6L)</td><td>35.7(1)</td><td></td><td>(H_6L)</td><td>53.9(1)</td><td></td><td>(H_6L)</td><td>59.7(1)</td><td></td></tr>
</table>

Data S2. Discussion of calculated pK_a values in Table S7 for the titration of apo-Ylip_MT with Cu(I)

The complex formation constants and the calculated pK_a values of Ylip_MT in the presence of Cu(I) ions are summarized in Table S7.

In the presence of 1 equivalent of Cu(I), two pK_a values decrease to 3.4. The pK_a for the Cu$(H_6L)H_7$ species is with 4.1 only slightly higher than the one calculated for C-terminal-Ylip_MT (3.8), suggesting that the respective ligand may still participate in Cu(I) coordination. The next pK_a value of 4.6 may be either assigned to a coordinating cysteine or alternatively to the cysteine that showed a lowered pK_a value already in the

apo-form (5.1). Overall, the pK_a values in the full-length protein are higher than those observed in the truncated variants. A possible explanation could be that the longer and more flexible N-terminal segment shields the cysteine-rich C-terminal domain from deprotonation. Molar absorptivity calculations indicate that, at this stage, approximately three cysteine thiolate ligands are involved in coordinating the first Cu(I) ion (Table S2), which aligns with the results of the potentiometry data.

In presence of 2 equivalents of Cu(I), a total of five cysteine thiolates shows pK_a values below ≤ 4.2. One additional value is as low as 5.0. In view of the overall trend of decreasing pK_a values upon Cu(I) binding, it seems unlikely that the low pK_a observed in the apo-form (5.1) would now show a higher value and accordingly, we assign the pK_a of 5.0 also to a coordinating cysteine thiolate. The resulting total number of six coordinating thiolates aligns again with the UV data under the assumption, that in presence of three equivalents of Cu(I), all cysteine thiolates are coordinated.

In presence of three equivalents of Cu(I), six pK_a values are below 3.6 and also the last three protonation states (probably corresponding to two cysteine residues and the N-terminal amino group) exhibit a marked decrease with pK_a values of 4.1, 4.1, and 4.2, respectively. This suggests that all cysteine thiolates are already interacting with Cu(I) at this stage, again aligning with the above interpretation of the UV data.

Upon addition of the fourth Cu(I) equivalent, all nine pK_a values decrease to ≤ 3.7. As the molar absorptivity increases further, this stage likely involves the transition of some thiolates from terminal coordination to a µ-bridging mode. The pronounced decrease in the pK_a of the N-terminal amino group in the Cu_3 and Cu_4 species has already been discussed in the main text in the potentiometric section for C-terminal-Ylip_MT.

However, it is important to note that the potentiometric data do not allow the unambiguous assignment of specific pK_a values to the N-terminal amino group. It remains equally plausible that the higher pK_a values correspond to thiolates, while the N-terminal amino group undergoes an even more significant decrease.

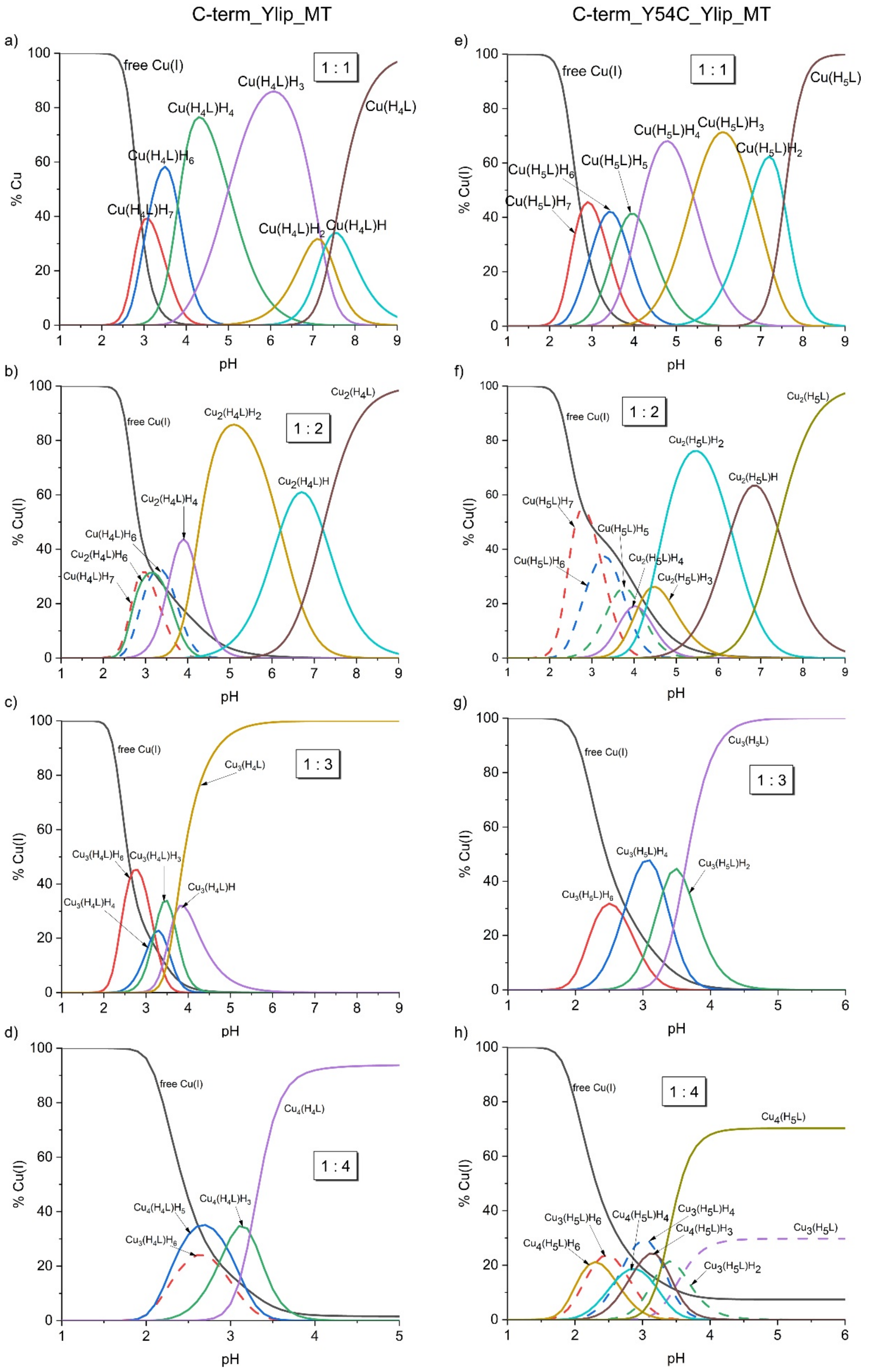

Figure S9. Representative species distribution diagrams for the Cu(I)-C-term_Ylip_MT (a-d) and C-term_Y54C_Ylip_MT (e-h) systems for peptide:Cu(I) molar ratios of 1:1, 1:2, 1:3, and 1:4. The distribution diagrams were calculated using the constants reported in Tables S5 and S6 and the program HySS2009.[3]

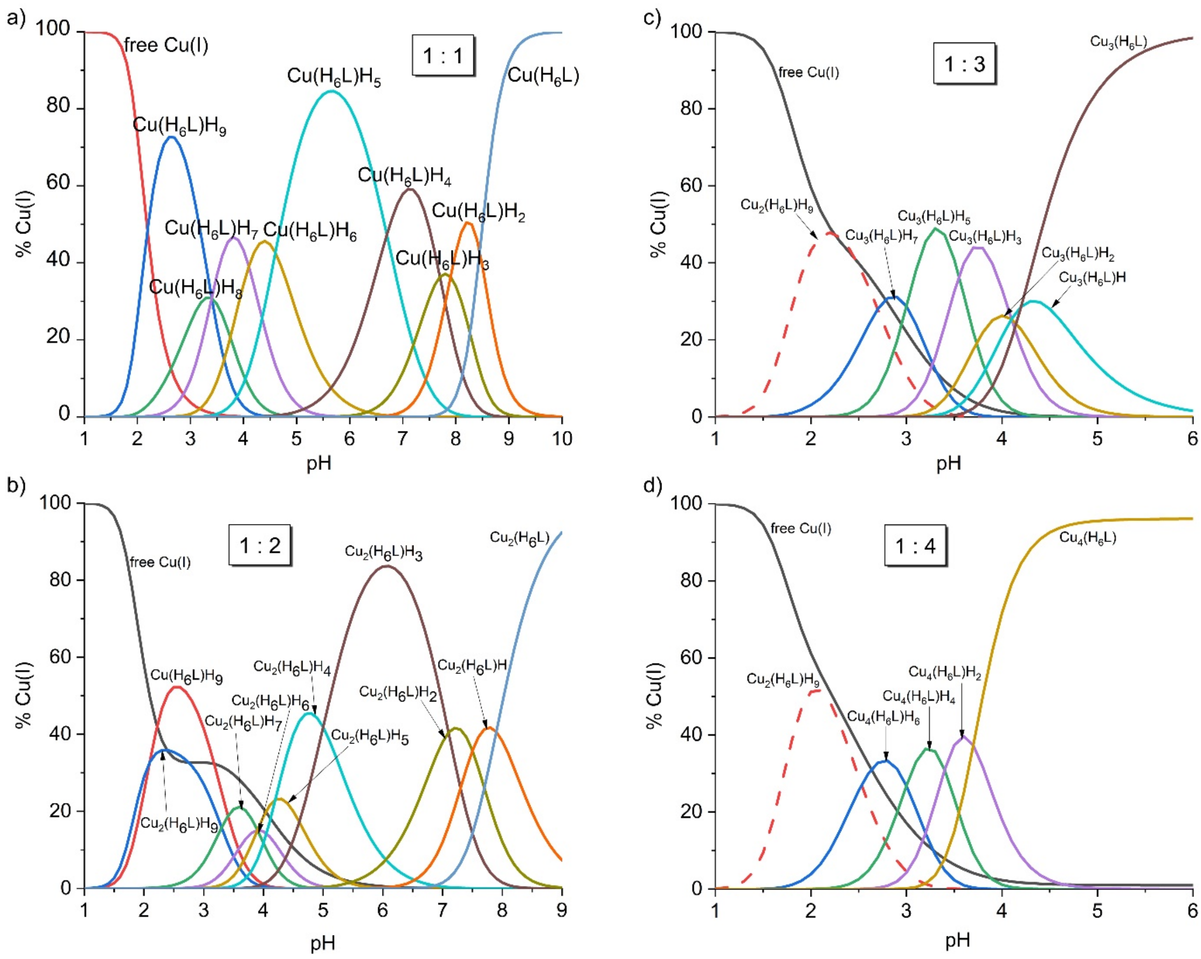

Figure S10. Representative species distribution diagrams for the Cu(I)-Ylip_MT system for peptide:Cu(I) molar ratios of 1:1, 1:2, 1:3, and 1:4. The distribution diagrams were calculated using the constants reported in Table S7 and the program HySS2009.[3]

Table S8. Analysis of the apo-Ylip_MT CD spectrum with the CDSSTR method in DichroWeb and different reference data sets.

Secondary structure	Set 3[4]	Set 4[4]	Set 6[4]	Set 7[4]	SP175[5]
Helix 1	0.02	-0.01	0.01	0.00	0.00
Helix 2	0.05	0.09	0.03	0.03	0.04
Strand 1	0.25	0.18	0.17	0.06	0.22
Strand 2	0.12	0.10	0.09	0.04	0.12
Turns	0.23	0.26	0.13	0.08	0.15
unordered	0.32	0.37	0.56	0.78	0.45
total	0.99	0.99	0.99	0.99	0.98
NRMSD	0.013	0.016	0.016	0.024	0.027

Table S9. Analysis of the apo-Y54C_Ylip_MT CD spectrum with the CDSSTR method in DichroWeb and different reference data sets.

Secondary structure	Set 3[4]	Set 4[4]	Set 6[4]	Set 7[4]	SP175[5]
Helix 1	0.00	-0.01	-0.01	-0.01	-0.01
Helix 2	0.02	0.07	0.01	0.01	0.04
Strand 1	0.23	0.21	0.25	0.15	0.24
Strand 2	0.13	0.13	0.13	0.09	0.14
Turns	0.20	0.23	0.20	0.15	0.13
unordered	0.40	0.36	0.40	0.58	0.44
total	0.98	0.99	0.98	0.97	0.98
NRMSD	0.047	0.032	0.051	0.036	0.046

Table S10. Analysis of the Cu_4Ylip_MT CD spectrum with the CDSSTR method in DichroWeb and different reference data sets.

Secondary structure	Set 3[4]	Set 4[4]	Set 6[4]	Set 7[4]	SP175[5]
Helix 1	-0.01	-0.01	0.00	-0.01	-0.01
Helix 2	0.01	0.05	-0.01	-0.01	-0.01
Strand 1	0.24	0.21	0.25	0.20	0.32
Strand 2	0.15	0.13	0.13	0.11	0.14
Turns	0.22	0.24	0.15	0.16	0.10
unordered	0.38	0.36	0.45	0.52	0.43
total	0.97	0.98	0.97	0.97	0.97
NRMSD	0.026	0.066	0.020	0.031	0.033

Table S11. Analysis of the Cu_4Y54C_Ylip_MT CD spectrum with the CDSSTR method in DichroWeb and different reference data sets.

Secondary structure	Set 3[4]	Set 4[4]	Set 6[4]	Set 7[4]	SP175[5]
Helix 1	0.09	0.00	0.01	0.02	0.06
Helix 2	0.09	0.12	0.05	0.04	0.10
Strand 1	0.16	0.16	0.21	0.16	0.14
Strand 2	0.11	0.09	0.11	0.09	0.10
Turns	0.22	0.27	0.20	0.24	0.15
unordered	0.35	0.36	0.40	0.44	0.45
total	1.02	1.00	0.98	0.99	1.00
NRMSD	0.007	0.010	0.013	0.009	0.014

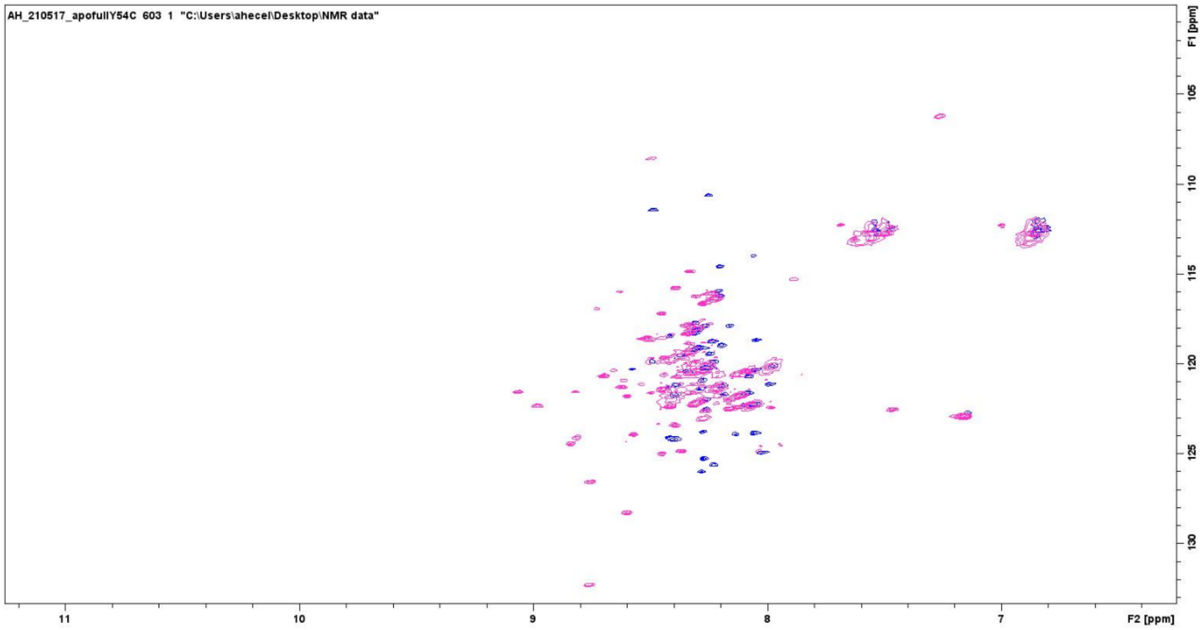

Figure S11. Overlay of [^{15}N, ^{1}H]-HSQC spectra of apo-Y54C_Ylip_MT (blue) and Cu_4Y54C_Ylip_MT (magenta).

References

1. R. Caspi, R. Billington, C. A. Fulcher, I. M. Keseler, A. Kothari, M. Krummenacker, M. Latendresse, P. E. Midford, W. K. Ong, S. Paley, P. Subhraveti and P. D. Karp, *FASEB J.*, 2019, **33**.
2. P. D. Karp, R. Billington, R. Caspi, C. A. Fulcher, M. Latendresse, A. Kothari, I. M. Keseler, M. Krummenacker, P. E. Midford, Q. Ong, W. K. Ong, S. M. Paley and P. Subhraveti, *Brief Bioinform.*, 2019, **20**, 1085-1093.
3. L. Alderighi, P. Gans, A. Ienco, D. Peters, A. Sabatini and A. Vacca, *Coord. Chem. Rev.*, 1999, **184**, 311-318.
4. N. Sreerama and R. W. Woody, *Anal. Biochem.*, 2000, **287**, 252-260.
5. J. G. Lees, A. J. Miles, F. Wien and B. A. Wallace, *Bioinformatics*, 2006, **22**, 1955-1962.
